# Synchrotron-Based Fourier-Transform Infrared Micro-Spectroscopy (SR-FTIRM) Fingerprint of the Small Anionic Molecule Cobaltabis(dicarbollide) Uptake in Glioma Stem Cells

**DOI:** 10.3390/ijms22189937

**Published:** 2021-09-14

**Authors:** Miquel Nuez-Martínez, Leire Pedrosa, Immaculada Martinez-Rovira, Ibraheem Yousef, Diouldé Diao, Francesc Teixidor, Elisabetta Stanzani, Fina Martínez-Soler, Avelina Tortosa, Àngels Sierra, José Juan Gonzalez, Clara Viñas

**Affiliations:** 1 Institut de Ciència de Materials de Barcelona, ICMAB-CSIC, Campus Universitat Autònoma de Barcelona, 08193 Bellaterra, Spain; mnuez@icmab.es (M.N.-M.); teixidor@icmab.es (F.T.); 2 Laboratory of Experimental Oncological Neurosurgery, Neurosurgery Service, Hospital Clinic de Barcelona—FCRB, 08036 Barcelona, Spain; lepedrosa@clinic.cat (L.P.); dioulde.dg@gmail.com (D.D.); jjgonzal@clinic.cat (J.J.G.); 3 Ionizing Radiation Research Group (GRRI), Physics Department, Universitat Autònoma de Barcelona (UAB), Avinguda de l’Eix Central, Edifici C. Campus de la UAB, 08193 Cerdanyola del Vallès, Spain; Immaculada.Martinez@uab.cat; 4 ALBA-CELLS Synchrotron, MIRAS Beamline, Carrer de la Llum 2-26, 08290 Cerdanyola del Vallès, Spain; iyousef@cells.es; 5 Laboratory of Pharmacology and Brain Pathology, IRCCS Humanitas Research Hospital, 20089 Rozzano, Italy; stanzani.elisabetta@gmail.com; 6 Apoptosis and Cancer Unit, Department of Physiological Sciences, IDIBELL, Faculty of Medicine and Health Sciences, Universitat de Barcelona, 08907 L’Hospitalet del Llobregat, Spain; finamartinez@ub.edu (F.M.-S.); atortosa@ub.edu (A.T.)

**Keywords:** boron neutron capture therapy, cobaltabis(dicarbollide), DNA interactions, glioblastoma, glioma initiating cells, inorganic small molecules, lipid saturation, SR-FTIRM

## Abstract

The anionic cobaltabis (dicarbollide) [3,3′-Co(1,2-C_2_B_9_H_11_)_2_]^−^, [*o*-COSAN]^−^, is the most studied icosahedral metallacarborane. The sodium salts of [*o*-COSAN]^−^ could be an ideal candidate for the anti-cancer treatment Boron Neutron Capture Therapy (BNCT) as it possesses the ability to readily cross biological membranes thereby producing cell cycle arrest in cancer cells. BNCT is a cancer therapy based on the potential of ^10^B atoms to produce α particles that cross tissues in which the ^10^B is accumulated without damaging the surrounding healthy tissues, after being irradiated with low energy thermal neutrons. Since Na[*o*-COSAN] displays a strong and characteristic ν(B-H) frequency in the infrared range 2.600–2.500 cm^−1^, we studied the uptake of Na[*o*-COSAN] followed by its interaction with biomolecules and its cellular biodistribution in two different glioma initiating cells (GICs), mesenchymal and proneural respectively, by using Synchrotron Radiation-Fourier Transform Infrared (FTIR) micro-spectroscopy (SR-FTIRM) facilities at the MIRAS Beamline of ALBA synchrotron light source. The spectroscopic data analysis from the bands in the regions of DNA, proteins, and lipids permitted to suggest that after its cellular uptake, Na[*o*-COSAN] strongly interacts with DNA strings, modifies proteins secondary structure and also leads to lipid saturation. The mapping suggests the nuclear localization of [*o*-COSAN]^−^, which according to reported Monte Carlo simulations may result in a more efficient cell-killing effect compared to that in a uniform distribution within the entire cell. In conclusion, we show pieces of evidence that at low doses, [*o*-COSAN]^−^ translocates GIC cells’ membranes and it alters the physiology of the cells, suggesting that Na[*o*-COSAN] is a promising agent to BNCT for glioblastoma cells.

## 1. Introduction

Glioblastoma (GBM) is the most frequent and aggressive primary tumor in the central nervous system (CNS). Owing to their fast-clinical course and uniform lethality after standard treatment—surgical resection followed by chemoradiotherapy regimen [1]—most patients with GBM have a median survival of approximately 15 months from diagnosis [2] because almost all GBMs develop resistance to therapy and recurrence [3,4].

In vitro and in vivo observations give strength to the existence of human glioblastoma cell subpopulations with the ability to initiate tumors, when injected orthotopically into mice [5,6]. These glioma initiating cells (GICs) express stemness-related markers and perform cellular niches supported by the tumor microenvironment recapitulating cellular heterogeneity, chemo- and radio-resistance [7]. 

Adult GBMs can be classified according to their gene expression and epigenetic profiles into four different subtypes: glioma-CpG island methylator phenotype (G-CIMP^+^) and three non-G-CIMP (G-CIMP^−^) subtypes termed as proneural (PN), classical (CL), and mesenchymal (MES) [8,9,10]. G-CIMP^+^ GBMs are associated with somatic mutations in the IDH1 or IDH2 genes and show a more favorable prognosis, whereas non-G-CIMP GBM patients have the poorest prognosis [8]. Non-G-CIMP GBMs display elevated transcriptional plasticity and have an intrinsic ability to transition from PN to MES phenotype, more aggressive, with a higher likelihood of recurrence [11] and therapy resistance [4,12].

In the last decade, boron neutron capture therapy (BNCT), a tumor-selective particle radiation therapy, emerges as an alternative treatment, when chemotherapy and radiation fell short [13]. Indeed, BNCT has been used to treat recurrent or newly diagnosed high-grade glioma showing prolonged survival about the control cohort [14]. This non-invasive binary cancer radiotherapeutic modality is based on the nuclear capture and fission reactions that occur when the stable isotope ^10^B, a constituent of natural elemental boron, is irradiated with low-energy epithermal neutrons (10,000 eV) to produce high linear-energy transfer α particles [15,16], which locally deposit the energy in tissues and thus, enhances the dose in tumor cells.

There are quite a few strict pre-requisites before employing boron drugs for BNCT applications regarding their toxicity, solubility, and on their ability to reach the required amount of ^10^B inside the target cells. It is accepted that 20–30 µg of ^10^B per gram of tumor is required for ensuring the therapeutic efficiency of BNCT [17]. With the development of new synthetic techniques and increased awareness of the biochemical requirements needed for effective boron-containing agents, several new neutral [18,19,20,21] and anionic metallacarborane compounds have been developed [22,23,24]. Among them, the anionic cobaltabis(dicarbollide) [3,3′-Co(1,2-C_2_B_9_H_11_)_2_]^−^, [*o*-COSAN]^−^, in which Co^3+^ ion is sandwiched between two [7,8-C_2_B_9_H_11_]^2−^ ligands is the most studied icosahedral metallacarborane. The abiotic icosahedral anionic [*o*-COSAN]^−^ cluster, which incorporates the redox properties of the metal, is thermally and chemically stable [24] with the negative charge spread all over the molecule [25], and possesses many possibilities to produce hydrogen and dihydrogen bonds (C_c_-H···O and C_c_-H···H-B or N-H···H-B, respectively; C_c_ represents the C_cluster_ atoms). The hydrogen and dihydrogen bonds have been proven to participate in its self-assembly [26,27,28], water solubility [29], as well as in its micelles and vesicles formation [29,30,31,32,33].

Recently, Synchrotron-based Fourier-transform infrared micro-spectroscopy (SR-FTIRM) has developed into a novel and powerful biomedical tool that can reveal subtle alterations in the conformation of key molecular structures of cells (DNA, proteins, and lipids) providing rapid, non-destructive, and clinically relevant diagnostic information [34]. SR-FTIRM, which couples IR spectrometer with single element detectors and optical microscope, has become a valuable technique for analyzing the vibrational motions on a microscopic scale of biomolecules [35]. This technique allows in situ structure determination of the most important biomolecules (nucleic acids, proteins, lipids) in the chosen sub-cellular compartments [36]. The higher spatial resolution and spectral quality, in comparison with the “classic” Fourier-transform infrared microscopy, can be attributed to the synchrotron IR source, which is 100–1000 times brighter than a conventional thermal source for IR spectroscopy [37]. The higher brightness (flux density) of the synchrotron that is due to the small effective source and its very narrow emittance, allows the analysis of smaller regions with an acceptable signal-to-noise ratio, and the spatial resolution reaches the diffraction limit (λ/2) [34,38,39]. The structure of biomolecules inside cells can be probed with a sub-cellular resolution thanks to the high spatial resolution of the SR-FTIR technique, which generates many data. The IR spectra collected of hundreds of cells need subsequently the application of multivariate statistical methods for efficient data analysis. The principal component analysis (PCA) is a powerful method for analyzing the large amount of spectral data set for the presence of discriminant features that classify the spectra [40,41].

Having performed experiments in a round-bottom flask on a chemical scale, which showed that [*o*-COSAN]^−^ and some of its halogenated derivatives interact with biomolecules (carbohydrates [42], proteins [43] and DNA [44]), we have wanted to observe these interactions in vitro experiments by changing the round-bottom flask to a cell and the solutions to the physiological components of the cell. In addition, the previous studies at the chemical scale were done individually, whereas the present study incorporates the effect of all the interacting biomolecules in a cell. Furthermore, we wanted to go a step ahead to understand if this small anion modifies some of the biomolecules and in which of the cells’ organelles the [*o*-COSAN]^−^ concentrates. With this in mind, in this study, we show that SR-FTIRM can be applied to the detection of boron clusters (boranes, carboranes, and metallacarboranes) interacting with biomolecules inside cells and their location into the cells’ organelle after their cellular uptake. This is possible because these compounds’ families display a strong and characteristic ν B-H frequency in the infrared range 2.600–2.500 cm^−1^ in which no other frequencies of organic compounds appear. With this objective, we studied the uptake of the sodium salt of cobaltabis(dicarbollide), [Na·2.5H_2_O][3,3′-Co(1,2-C_2_B_9_H_11_)_2_], abbreviated as Na[*o*-COSAN], in two different phenotypes of glioma initiating cells (GICs), mesenchymal- and proneural- GICs, using SR-FTIR micro-spectroscopy to analyze where the agent is located into the cells after their cellular uptake.

Infrared micro-spectroscopy has been used to investigate many phenomena in biology, but in this work we demonstrate for the first time that it can differentiate the two phenotypes of glioma initiating cells (GICs), mesenchymal and proneural GICs, mainly by DNA spectra, protein conformation and lipids components, which were differently modified by Na[*o*-COSAN]. Interestingly, specific changes in lipid 2966 cm^−1^ and 2922 cm^−1^ frequencies are induced in radio resistant mesenchymal cells PG88, the most efficient in capturing the compound. Although more experiments are needed including representative GICs from glioblastoma phenotypes, these results suggest that SR-FTIRM is useful to acquire a drug and radio-resistance profiling of glioblastoma cells. Moreover, the high uptake of Na[*o*-COSAN] with rapid clearance might fulfil the requirements of boron-containing agents. Furthermore, we also pioneer that SR-FTIRM can be of great interest for BNCT studies. The vast majority of compounds tested for BNCT contain the B-H bond and the IR frequency corresponding to this bond appears in an area of the spectrum where no other signal is observed, thus leaving conclusive evidence of its presence in the tumor or healthy cells under study. This work is therefore dedicated to testing the possibilities of this spectroscopy to study the behavior of a metallacarborane both in its permeation process into the cell and in its irregular distribution in the cell depending on the composition of the site, be it DNA, proteins or lipids. 

## 2. Results and Discussion

### 2.1. o-Cobaltabis(Dicarbollide), [o-COSAN]^−^ Uptake Induces Glioma Initiating Cells (GICs) Phenotypic Changes

**Figure 1:**
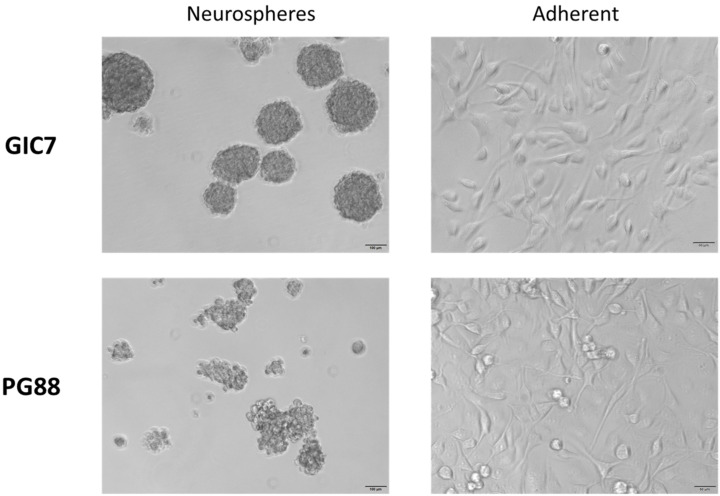
Morphology of glioblastoma initiating cells (GICs). Proneural GIC7 (top) and mesenchymal PG88 (bottom) cells grow performing neurospheres (**left**, 4×, scale bar 100 μm) or adhered on laminin-coated plates (**right**, 10×, scale bar 50 μm) when cultured in a complete Neuronal Stem Cell medium at 37 °C in a humidified 5% CO_2_ and 5% O_2_ atmosphere (hypoxia conditions) to simulate the brain microenvironment.

**Figure 2:**
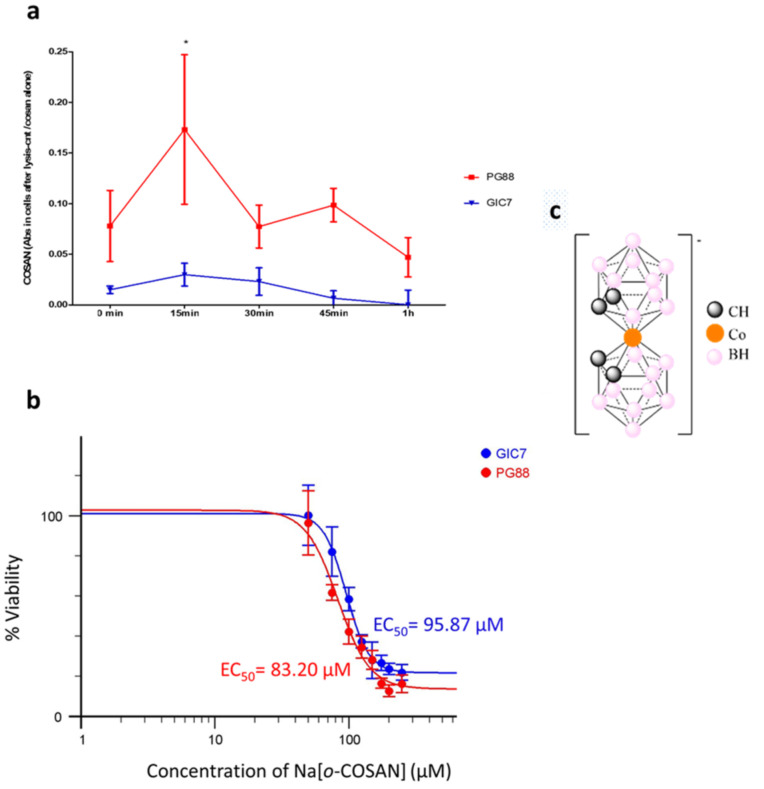
Kinetic of Na[*o*-COSAN] uptake in glioma undifferentiated cells (GICs). Proneural (GIC7) and mesenchymal (PG88) GICs cultured on laminin-coated wells, 300,000 GICs/well, were seeded 24 h before treated with Na[*o*-COSAN] (200 µM). (**a**) Time course analysis of Na[*o*-COSAN] uptake in lysed cells from GIC7 (blue) and PG88 (red) respective cultures are represented from Spectrometric Na[*o*-COSAN] data analyzed (Synergy), plotted as the percentage of untreated cell cultures, mean ± SD, of three independent experiments. The uptake of the compound increased at 15 min of treatment on both GICs, being higher in PG88 concerning GIC7 (ANOVA, * *p* < 0.05). (**b**) Na[*o*-COSAN] half-maximal effective concentration (EC_50_) at 48 h in a doses-response curve, ranged 50 to 300 µM. EC_50_ of GIC7 (95.87 µM) and PG88 (83.2 µM) cells are not significantly different. (**c**) Schematic representation of the anionic small molecule cobaltabis (dicarbollide), [3,3′-Co(1,2-C_2_B_9_H_11_)_2_]^−^, abbreviated as [*o*-COSAN]^−^.

Analogous to whole tumors and according to GBM-subtype, glioblastoma isolated tumor cell produces tumorspheres enriched in glioma stem-like initiating cells (GICs) with specific phenotypes (Figure 1). Since previous studies indicated that Na[*o*-COSAN] enters and accumulates into glioma U87 cells [45], which can be detected by one absorption band λ_max_ at 282 nm (ε ≈ 30.000 cm^−1^ mol^−1^) in the UV-Visible spectra, we analyzed Na[*o*-COSAN] uptake in GICs from two different glioblastoma subtypes: proneural-GIC (GIC7) and a mesenchymal-GIC (PG88), that were previously characterized by their different radiosensibility [4,46]. Schematic representation of the anionic small molecule [*o*-COSAN]^−^ is displayed in Figure 2c.

Laminin-coated GICs’ exposed at 200 µM Na[*o*-COSAN] were analyzed at 282 nm wavelength using UV-visible spectroscopy. To assess cellular uptake we measured the optical density of adhered cells at different exposition times: 0, 15, 30, 45, and 60 min. The ratio between the absorbance (Abs) of treated cells to the untreated controls were calculated for each time-point (Figure 2). Abs was recorded at 282 nm wavelength, which corresponded to the maximum λ of [*o*-COSAN]^−^ [44,47,48].

Na[*o*-COSAN]^−^ has the unusual property of self-assembling into vesicles on biological cell membranes [29]. Thus, Na[*o*-COSAN] levels increased in the intracellular compartment of GIC7 and PG88 cells at 15 min (Figure 2a). After 30 min, the concentration of [*o*-COSAN]^−^ increased in cellular extracts in both the cell types. These results indicated that Na[*o*-COSAN] can cross the cellular membrane and enter the cells in a short period, suggesting a fast transport from the extracellular compartment. Moreover, Na[*o*-COSAN] uptake was higher in PG88 than in GIC7 cells (0.17 nm and 0.03 nm, respectively) at 15 min (two-way ANOVA and Bonferroni posttests, *p* < 0.05), and differences between both cells were maintained until the end of the experiment one hour later (Figure 2a), when the intracellular levels of the compound decreased in both (0.047 and 0.0002 nm, respectively). Altogether, we concluded that a fast Na[*o*-COSAN] internalization occurred in both, mesenchymal and proneural cells, with mesenchymal PG88 cells being the most efficient in capturing the compound.

The dynamic profiling of Na[*o*-COSAN] is crucial in BNCT, based on the nuclear capture and fission reactions that occur when the stable ^10^B isotope is irradiated with epithermal neutrons (10,000 eV) and become thermalized as they penetrate tissue [16]. The destructive effects of the α particles are limited to the ^10^B atoms of the boron-containing cells and, because they have very short path lengths in tissues (5–9 μm), BNCT provides a way to selectively destroy malignant cells and spare surrounding normal tissue, making it, in theory, an ideal type of radiation therapy [15].

We analyzed the Na[*o*-COSAN] half-maximal effective concentration (EC_50_) in a doses-response curve at 50 to 300 µM range concentration. EC_50_ values were calculated using exponential curve fitting. Controls without Na[*o*-COSAN] were considered 100% of viability to evaluate and graphically represent using the https://www.aatbio.com/tools/ec50-calculator program (accessed on 10 March 2020). (Figure 2b).

The viability assay measured at 43 h after treating the cells for five hours showed that EC_50_ was lower in PG88, 83.2 ± 9.652 µM (95% confident intervals: 62.99 to 102.7), than in GIC7 cells, 95.87 ± 7.592 µM (95% Confidence Intervals: 80.52 to 111.7), suggesting that GIC7 cells were more resistant than PG88 cells when challenged with [*o*-COSAN]^−^ (ANOVA *p*-value 0.0023). 

Since mesenchymal PG88 cells are more resilient than proneural GIC7 cells to conventional radiotherapy, a lower EC_50_ Na[*o*-COSAN] together with higher uptake of the compound in PG88 cells compared to GIC7 cells suggest a new resource to fight against resistant glioblastoma cells. Moreover, both GICs are more susceptible to Na[*o*-COSAN] based therapy than other cancer cells, in which preliminary studies showed that Na[*o*-COSAN] EC_50_ of 3T3 fibroblast cells was 99 ± 5.5 µM, while the most resistant HeLa cells had a Na[*o*-COSAN] EC_50_ of 157 ± 8.6 µM. Hence, we suggest that Na[*o*-COSAN] might be a new glioblastoma potential therapeutic compound to be used in BNCT. 

More experiments are needed to understand the biological reason of these kinetics variations between GIC7 and PG88 glioblastoma cells. It was demonstrated that the major problem with boron compounds is the significant variability in tumor uptake, especially in brain tumors. It has been calculated that, in order to be effective, approximately 20–30 µg of ^10^B per gram of tumor, which corresponds to 10^9^ atoms of ^10^Boron must be selectively delivered to individual cancer cells [17]. 

The pro-inflammatory environment induced by glioblastoma radiation therapy drives oncogenic signaling that confers radio resistance and activates mechanisms involved in the progression of proneural cells to a more aggressive mesenchymal phenotype with a more likelihood of disease relapse [49]. Therefore, BNCT appears as an attractive alternative to treat recurrent mesenchymal glioblastoma that has developed post-surgical photon irradiation resistance. 

**Figure 3:**
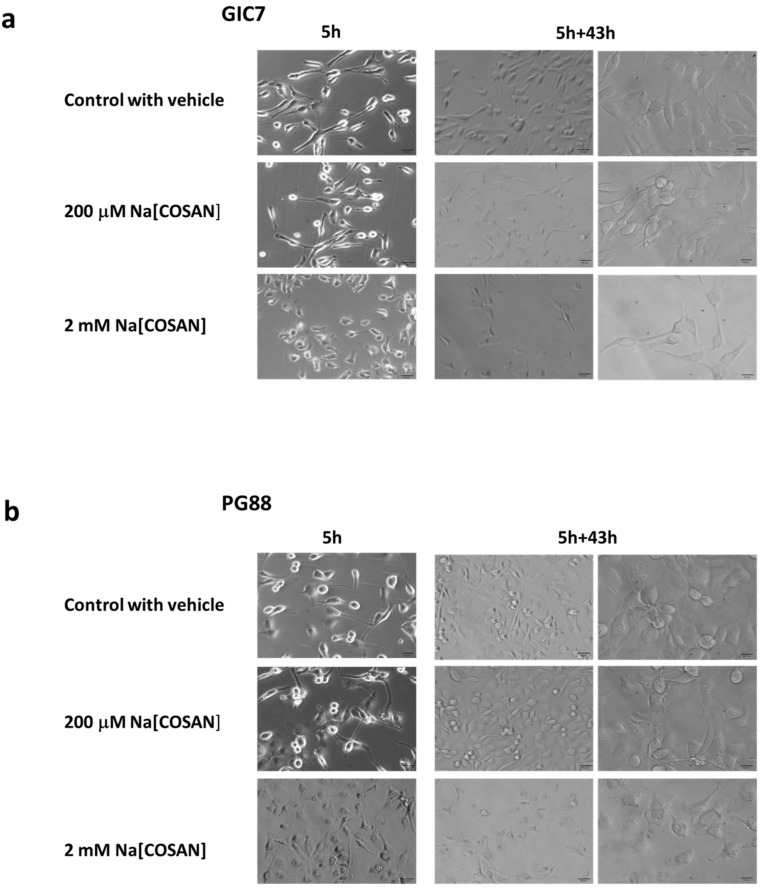
Morphology of glioblastoma initiating cells (GICs) treated with Na[*o*-COSAN]. Microscopy comparative analysis at 5 h treatment with Na[*o*-COSAN] 200 µM and 2 mM (left panels of (**a**,**b**) concerning untreated cells, and 43 h after washing out Na[*o*-COSAN] (×10 and ×20, middle and right panels magnificence, scale bars 50 μm in left and middle and and 25 μm, in right). Morphological changes on both GICs appear at 2 mM. GIC7 looks less affected than PG88 after 2 mM treatment and both cells show similar recovery after washing out the compound.

Na[*o*-COSAN] elicits a range of cell biological effects, including altered cell morphology, inhibition of cell growth, and, in some cases, apoptosis [45]. We checked morphological changes of both GICs, PG88 and GIC7, by microscopy after 5 h of treatment of 200 µM and 2 mM Na[*o*-COSAN] (Figure 3). The shape of cells turned in less spicular and brightness suggesting that cell death started in both GIC7 (Figure 3a) and PG88 (Figure 3b) cells. This morphology reverted 43 h after Na[*o*-COSAN] was removed and replaced by fresh complete culture medium as the cells recovered their usual morphological appearance, similar to control cells without the compound. Even when we treated cells with 2 mM Na[*o*-COSAN], which induced intense changes on GIC7 and PG88 cells at 5 h treatment, the remaining cells recovered their shape 43 h after washing out the compound (Figure 3a,b), with PG88 cells being the most affected. These results suggested that in both glioblastoma cells, a transient cytostatic effect is induced by Na[*o*-COSAN] instead of cytotoxicity, compatible with compound accumulation within cells, without affecting membrane integrity. Similar results were found previously in HeLa cells that showed an unusual, highly vacuolated morphology within the perinuclear cytoplasm when grown in the presence of Na[*o*-COSAN] [50], that reverted to their native morphology seen in untreated cells, at either 5 and 12 h after washout [*o*-COSAN]^−^. Moreover, Plesek reported that most of the polyhedral boranes are essentially nontoxic due to their inertness to biochemical reactions [51]. Later, some of the authors demonstrated [52] that the protonated and sodium salts of [*o*-COSAN]- possess the ability to readily cross biological membranes [52,53] even being negatively charged and accumulate inside living cells with no appreciable effect on cell viability. Since [*o*-COSAN]- is non- cytotoxic [51], but is cytostatic over long term, and cells are recovered following its removal [50], we can state that the results are consistent with others in the literature.

**Figure 4:**
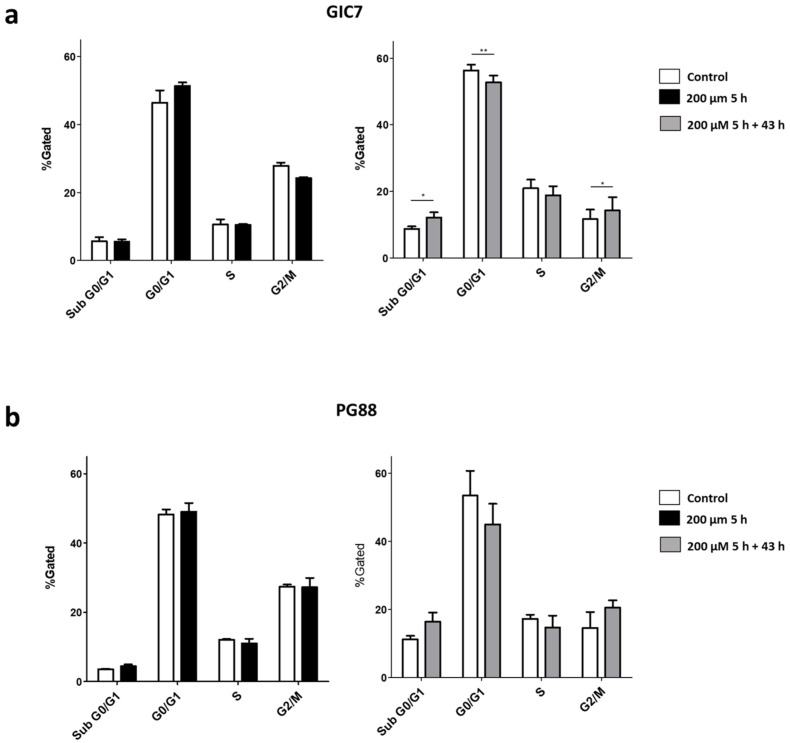
GIC7 and GP88 cell cycle changes induced by 200 µM Na[*o*-COSAN] 5 h treatment. Scheme 7. (**a**) nor PG88 (**b**) concerning their respective untreated controls. (**a**) GIC7 cell cycle at 43 h after washing out the Na[*o*-COSAN] (right panel) shows changes of sub G_0,_ G0/G1 and G2/M statistically significant (ANOVA, * *p* < 0.05; ** *p* < 0.01, respectively) (**b**) GP88 cell cycle at 43 h after wash out the Na[*o*-COSAN] (right panel) shows some differences in G0/1, G2/M and S phase not statistically significant.

We analyzed by flow cytometry the cell-cycle process that consists of four distinct ordered phases: G_0_/G_1_ (gap 1), DNA synthesis, G_2_ (gap 2), and M (mitosis), highly conserved and precisely controlled to govern the genome duplication. GICs treated with 200 µM Na[*o*-COSAN] 5 h showed no differences concerning control cells, neither GIC7 nor GP88, (Figure 4a,b, left). When GIC7 cells were morphologically recovered, 43 h after washing off the compound, we found a decreased G_0_/G_1_ subpopulation (two-way ANOVA and Bonferroni posttests, *p* < 0.01), about untreated control cells (Figure 4a right) and appeared a sub-G_0_ population (two way ANOVA and Bonferroni posttests, *p* < 0.05) compatible with cells dead, whereas the increase in G_2_/M (two way ANOVA and Bonferroni posttests, *p* < 0.05) subpopulation suggested the recovery of cells engaging a new cell cycle round. Similar results, statistically insignificant, were also found in PG88 cells (Figure 4b right).

Since the G_1_ and G_2_ phases are critical regulatory checkpoints [54], whereby the restriction point between the G_1_ and S phase determines whether the cells enter the S phase or exit the cell cycle, cells recovering from Na[*o*-COSAN] effect are halted at the G_0_ phase and a small percentage died. When cells are recovered, 43 h after treatment, they might enter in G_2_/M phase to complete division. Consequently, these results indicated that Na[*o*-COSAN] has a cytostatic effect on both GIC7 and PB88 cells with an increased recovery of GIC7 over PG88, suggested by the differences between them on G_2_/M, and probably due to the higher EC_50_ of GIC7 cells. 

### 2.2. GICs Na[o-COSAN] Uptake Analysis by SR-FTIRM and Multivariate Analysis

Synchrotron-based Fourier-transform infrared micro-spectroscopy (SR-FTIRM), which is a non-destructive methodology with minimal sample’s preparation and no requirement of radioactive labeling, facilitates the acquisition of biochemical signatures from cellular material (DNA, proteins, and lipids) based on the intrinsic molecular vibrations of the chemical bonds present [55]. SR-FTIRM is one of the fastest tools to get important information about the chemical compounds in the molecular and sub-molecular structure of living cells. It provides the possibility to perform a qualitative and quantitative analysis at the molecular level. Biological samples include a mixture of proteins, nucleic acids, lipids, and carbohydrates; all these biochemical contents have specific absorption bands in the infrared frequency domain. In SR-FTIRM, the radiation is absorbed by the sample at specific frequencies corresponding to their chemical bond inherent vibrational mode. A change in the chemical environment of a bond can result in peak shifts and/or bands intensity change. A single base discrimination difference in oligonucleotides sequences of DNA has been previously distinguished [56]. As SR-FTIR spectroscopy typically gives rise to hundreds of wavenumbers per spectral acquisition, multivariate analysis is required because of the large volume of data acquired. Such multivariate analysis can be performed with principal component analysis (PCA), which facilitates unsupervised data reduction on the processed spectra [56]. Scores’ plots provide a visual interpretation of the spread of data and cluster vector plots allow identification of the wavenumbers responsible for segregation and any similarities or differences between classes.

The potential of using spectroscopic techniques for the analysis of DNA extracted from cells or tissues could enable new insights into diseases related to epigenetic events. The main objective of this study was to investigate by SR-FTIR micro-spectroscopy the interaction of [*o*-COSAN]^−^ anion with the different groups of cell’s biomolecules (lipids, proteins, and DNA) after the [*o*-COSAN]^−^ uptake by two GICs, PG88 and GIC7. Subsequently, we analyzed:

(i) IR spectra of Na[*o*-COSAN] at GICs.

**Figure 5:**
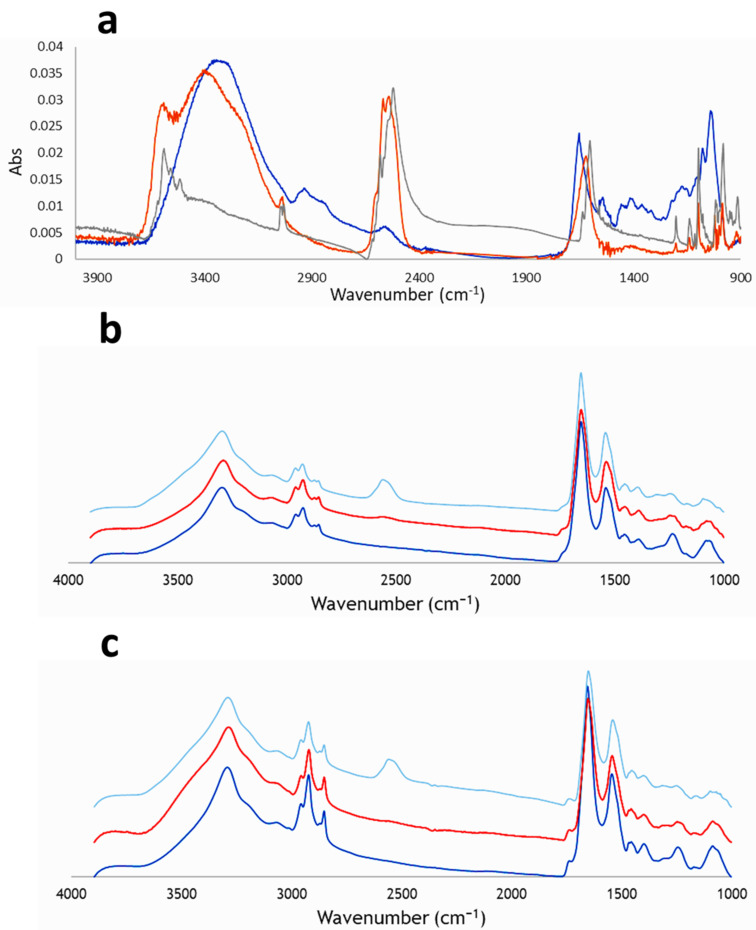
GICs Na[*o*-COSAN]^−^ uptake analyses by SR-FTIRM. Na[*o*-COSAN] spectra display a strong and characteristic ν(B-H) frequency at the infrared range 2.600–2.500 cm^−1^ in which no other frequencies of organic compounds appear. The IR spectrum of Na[*o*-COSAN] in solid-state displays the ν(B-H) and ν(C_c_-H) frequencies at 2582–2522 and at 3031 cm^−1^, respectively. (**a**) IR spectra of Na[*o*-COSAN] recorded in solid-state (grey), 2 mM of Na[*o*-COSAN] in aqueous solution (orange) and 2 mM of Na[*o*-COSAN] in culture media solution (blue). (**b**) IR spectra of GIC7 cells: control cells (dark blue), cells incubated with 200 µM of Na[*o*-COSAN] for 5 h (red) and, cells incubated with 2 mM of Na[*o*-COSAN] for 5 h (light blue). (**c**) IR spectra of PB88 cells: control cells (dark blue), cells incubated with 200 µM of Na[*o*-COSAN] for 5 h (red) and, cells incubated with 2 mM of Na[*o*-COSAN] for 5 h (light blue).

The first step of this study was to acquire the IR spectrum of Na[*o*-COSAN] in solid-state and in 2 mM solution of water and NSC medium to analyze the influence of both components, H_2_O and NSC medium, on the Na[*o*-COSAN] IR spectrum (Figure 5) and take them as reference for the GICs SR-FTIR spectroscopy. To emphasize that all three Na[*o*-COSAN] spectra display a strong and characteristic ν(B-H) frequency in the infrared range 2.600–2.500 cm^−1^ in which no other frequencies of organic compounds appear. The IR spectrum of Na[*o*-COSAN] in solid-state (Figure 5a, grey) displays the ν(B-H) and ν(C_c_-H) frequencies at 2582–2522 and 3031 cm^−1^, respectively. Bands at 3590, 3561, and 3518 cm^−1^ correspond to H_2_O coordinated to the Na cation. The IR spectrum of 2 mM aqueous solution of Na[*o*-COSAN] (Figure 5a, orange) is similar to the one in solid-state, but non-coordinated water from the solvent is also observed, while the IR spectrum of 2 mM solution of Na[*o*-COSAN] in the culture media (Figure 5a, blue) is different. In the latest spectrum, the frequencies related to the H_2_O and ν(C_c_-H) are not observed by visual examination and the peak intensity of the signal related to the ν(B-H) decreases; it is a clear indication of an alteration in the chemical environment of the B-H and C_c_-H bonds, which can be related to the interaction between Na[*o*-COSAN] and the nutrient biomolecules (proteins and amino acids) present in the NSC culture media.

(ii) Na[o-COSAN] uptake and localization on GICs.

To analyze the changes induced by the uptake of Na[*o*-COSAN] into the GIC cells, each cells line was incubated in a complete culture medium containing 200 µM or 2 mM of Na[*o*-COSAN] for 5 h. Further, the two cell lines were studied using SR-FTIR micro-spectroscopy. Three different samples per cell line were analyzed, recording the spectra of 100–200 cells in each sample. The experiments were performed as described in the methods section. Once the spectra were recorded, they were statistically analyzed separately at the lipids region and fingerprint region, which includes mainly proteins and DNA, by using PCA.

Well-defined peaks in the SR-FTIR averaged spectra were observed in treated GICs with Na[*o*-COSAN], either GIC7 or GP88, compared with the corresponding cell’s control (Figure 5b). The B-H stretching signal from the Na[*o*-COSAN] appears at 2557 cm^−1^ with a sub-peak at 2537 cm^−1^. The intensity of the IR peak is proportional to the amount of compound. As seen in Figure 5b, the peak from B-H signal observed in GIC7 cells after 200 µM Na[*o*-COSAN], 5 hours’ treatment, was small but detectable (Figure 5b,c, orange), but increased in both cell lines at 2 mM Na[*o*-COSAN] (Figure 5b,c, light blue).

(iii) Study of the SR-FTIR spectra of GICs treated with Na[o-COSAN] 5h.

The IR spectra were measured in the wavenumber range 4000–800 cm^−1^. We have studied separately the range region of the different cell components (DNA, proteins, and lipids) in the IR spectrum, which corresponds to 1350–900, 1800–1480, and 3000–2800 cm^−1^, respectively. Nucleic acids and sugars appear in the region 1350–900 cm^−1^ in which the symmetric and antisymmetric vibrations of the phosphodioxy [(RO)_2_PO_2_]^−^ groups appear [57]. The range 1800–1480 cm^−1^ corresponds to amide I vibrations (due to C=O and C-N stretching bands) and amide II vibrations (due to N-H bending and C-N stretching bands). The mean cellular contributors in the range 3000–2800 cm^−1^ correspond to fatty acids and lipids because of the alkyl chain stretching vibrations while the deformation vibrations of their CH_2_ and CH_3_ alkyl groups appear in the area 1480–1300 cm^−1^ [58].

**Figure 6:**
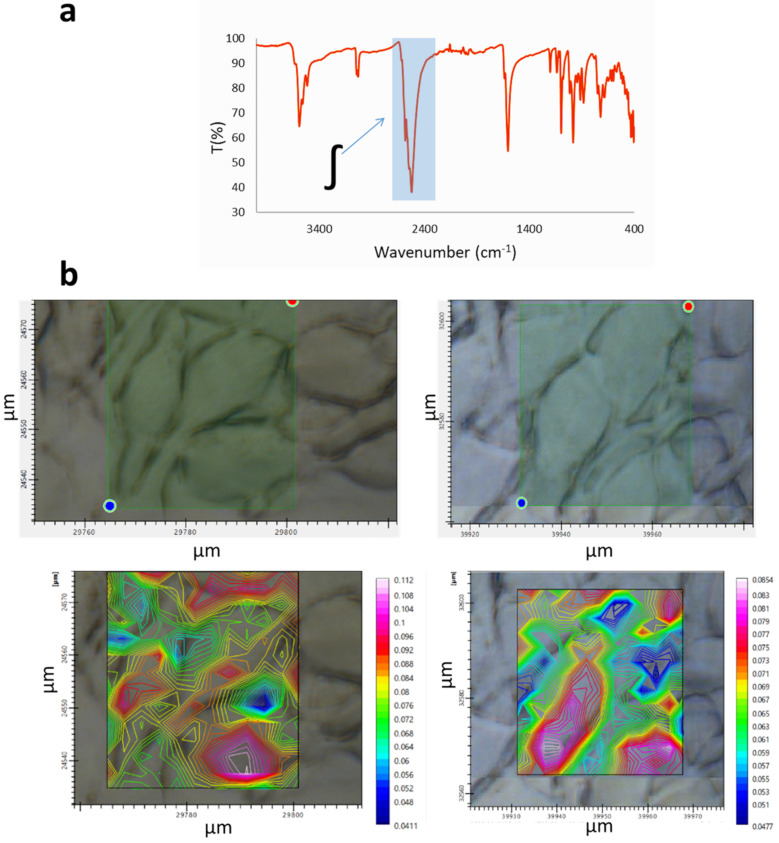
Microscopy images of GIC7 cells treated with Na[o-COSAN] 2 mM, 5 h and mappings were obtained by integrating the ν(B-H) signals between 2620 and 2460 cm^−1^ spectra (**a**). Two different areas of culture cells are shown with the corresponding maps (**b**). The red color intensity is proportional to Na[*o*-COSAN] spectra suggesting the nuclear localization of the compound.

We performed microscopy images of GIC7 cells treated with Na[*o*-COSAN] 2 mM and, 5 h to assess the intracellular location of the compound. Mappings were acquired by integrating the ν(B-H) signals between 2620 and 2460 cm^−1^ spectra in different areas of the culture. The analysis showed cell maps in which the color intensity was proportional to Na[*o*-COSAN] concentration (Figure 6). This mapping suggested the nuclear localization of the compound. These experimental results agree with the reported theoretical study, which found that hydrophobicity and the negative charge of nanoparticles are necessary for their translocation through the nuclear pore [59].

Moreover, Monte Carlo simulations showed that the amount of boron for BNCT should be localized preferably within or near the nucleus because the energy deposition in the nucleus of cells exposed to the neutron reaction results in a more efficient cell-killing effect compared to that in a uniform distribution within the entire cell [60]. 

In summary, cell mapping was acquired to determine the uptake of Na[*o*-COSAN], and spectroscopic data were obtained from bands in the regions that corresponded to the DNA, proteins, and lipids, which suggest that Na[*o*-COSAN] interacts with DNA strands, modify proteins structure, and lipids saturation, inducing changes in the lipid-protein ratio. These data suggest that Na[*o*-COSAN] is a real and new promising anionic inorganic small molecule candidate that might be used in BNCT of glioblastoma cells.

#### 2.2.1. SR-FTIRM at the DNA Region

**Figure 7:**
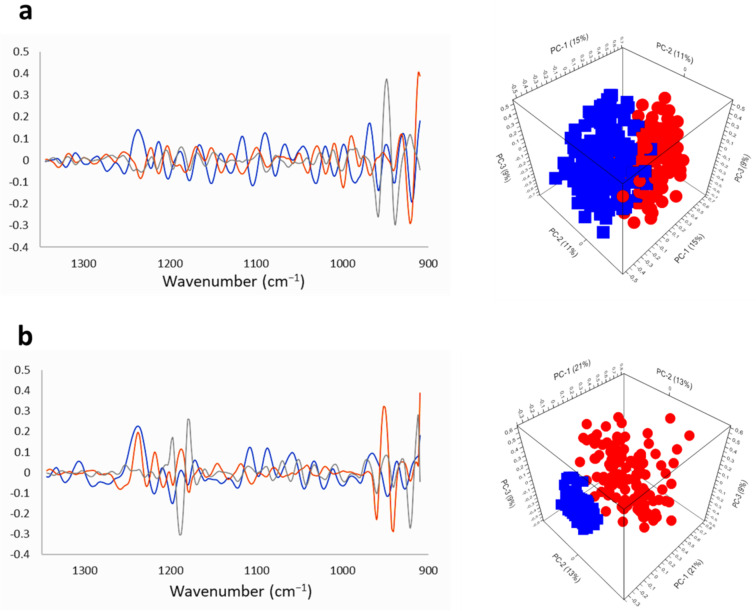
PCA and loadings within the DNA region (1350–900 cm−1) of GICs samples treated with Na[*o*-COSAN] 200 μM, 5 h: (**a**) GIC7 and (**b**) PG88 cells. Principal component loadings (blue PC-1, orange PC-2, grey PC-3) are shown on left panels. Right-panels principal component analysis score plots of PC-1, PC-2 and PC-3 displaying the variance between GIC control cells (blue) and GIC treated cells (red).

To analyze the influence of Na[*o*-COSAN] on DNA components of the two GIC cell lines, the PCA of the SR-FTIRM spectra was performed using the 1350–900 cm^−1^ region spectra second derivative (Supplementary Figure S1a,b). Several differences were observed between GIC7 control and GIC7 treated with 200µM Na[*o*-COSAN] (Figure 7a). Among others, the treatment decreased the intensity and the shape of the phosphate peak at 1250 cm^−1^ with regard control cells. Similar changes were also observed in PG88 cells (Figure 7b) and in both GICs treated with 2 mM Na[*o*-COSAN] (Supplementary Figure S2a,b).

Na[*o*-COSAN] induced spectral changes at the DNA region in both GIC cell lines, which are interestingly similar to the changes induced by other metal-based compounds like cisplatin that disrupt the double helix base pairing [61]. These results with Na[*o*-COSAN] go in parallel with previously reported studies that indicated metallabis (dicarbollide)’s strong interactions with DNA; the interaction is intercalative (in between both chains) in the case of Na[*o*-FESAN] [62] but electrostatic with Na[*o*-COSAN] [45]. In addition, previous SR-FTIRM studies on several human cancer cell lines unveiled specific spectral biomarkers of the drug interplay with the DNA (e.g., ν(OPO) and δ(NH_2_)) both through direct coordination to the double helix and via interference with the surrounding water molecules [63]. Variations were evidenced in DNA’s typical features, particularly those assigned to the phosphate groups. Therefore, these results suggest that the therapeutic action of [*o*-COSAN]^−^ might be based on interacting with DNA molecules of the GICs cells.

#### 2.2.2. SR-FTIRM at the Region of the Protein

**Figure 8:**
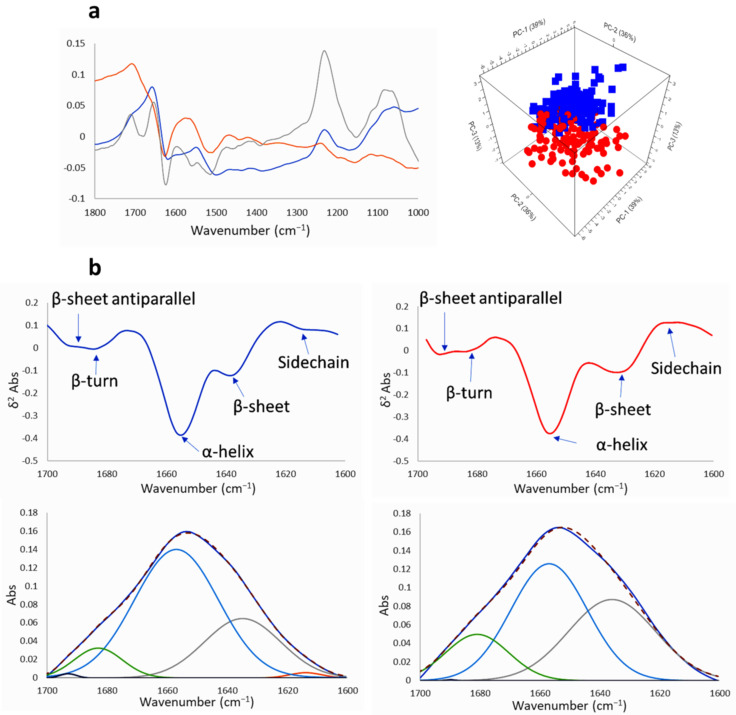
PCA and loadings within the protein region (1800–1000 cm^−1^) of GIC7 cells treated with Na[*o*-COSAN] 200 µM, 5 h: (**a**) Principal component loadings (blue PC-1, orange PC-2, grey PC-3) and principal components analysis scores plot of PC-1, PC-2 and PC-3 displaying the variance between GIC7 control (blue) and GIC7 treated cells (red). (**b**) The second derivative of FTIR spectrum at Amide I region 1700–1600 cm^−1^ and its deconvolution is shown to visualize differences between GIC7 untreated control (left) and GIC7 treated cells (right). The deconvolutions follow the following code: Amide I-experimental in dark blue, Sidechain in orange, β-sheet in grey, α-helix in blue, β-turn in green, β-sheet antiparallel in black, and Amide I-calculated in red dotted.

**Figure 9:**
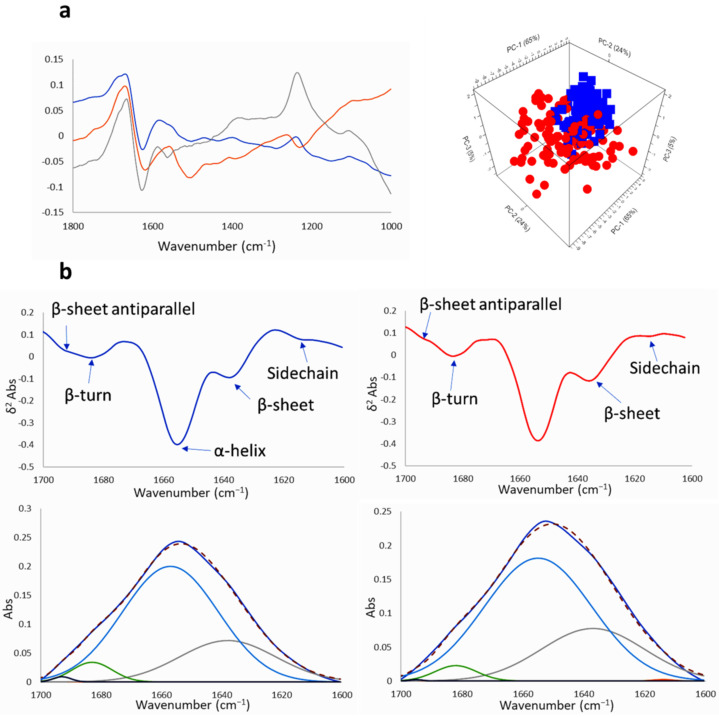
PCA and loadings within the protein region (1800–1000 cm^−1^) of PG88 cells treated with Na[*o*-COSAN] 200 µM, 5 h: (**a**) Principal component loadings (blue PC-1, orange PC-2, grey PC-3) and principal components analysis scores plot of PC-1, PC-2 and PC-3 displaying the variance between PG88 control (blue) and PG88 treated cells (red). (**b**) The second derivative of FTIR spectrum at Amide I region 1700–1600 cm^−1^ and its deconvolution is shown to visualize differences between PG88 untreated control (left) and PG88 treated cells (right). The deconvolutions follow the following code: Amide I-experimental in dark blue, Sidechain in orange, β-sheet in grey, α-helix in blue, β-turn in green, β-sheet antiparallel in black, and Amide I-calculated in red dotted.

The absorptions in the spectral range 1800–1350 cm^−1^ are due to the amide I and amide II vibrations of the proteins (Figure 8 and Figure 9 and Figures S3–S5). To explore the general variation induced by the compound, rubber band baseline correction and vector normalization was applied to raw spectra from the corresponding PCA of this fingerprint region, corrected by extended multiplicative signal correction (EMSC) of control untreated GICs vs. treated GICs with Na[*o*-COSAN] 200 µM for 5 h. Changes of protein profile were observed at 1522 cm^−1^ and 1641 cm^−1^ regions. Component PC-2 and PC-3 from PCA analyses of both GICs, GIC7 (Figure 8a) and PG88 (Figure 9a), are different between treated and non-treated cells. Changes in peak shape, absorbance, and peak position of Amides I 1650 cm^−1^ and 1550 cm^−1^ were observed. Moreover, at PC-2 the 1666 cm^−1^ peak Amide I increased on treated PG88 concerning the untreated control cells. In addition, PC-3 included strong changes in Amide I, 1650 cm^−1^, and DNA, 1250 and 1050 cm^−1^ peaks. 

The amide I signals were deconvoluted [64] to determine how Na[*o*-COSAN] affects the secondary structure of proteins (Figure 8b and Figure 9b) in both GIC cell lines, GIC7 and PB88. The peaks for the deconvolution were assigned using the 2nd derivative spectrum (Figure 8b and Figure 9b), where five bands were assigned to β-sheet antiparallel (at 1693 cm^−1^), β-turn (at 1683 cm^−1^), α-helix (at 1654 cm^−1^), β-sheet (at 1637 cm^−1^) and sidechain (at 1614 cm^−1^). The percentage area of α-helix and β-sheet structure of both GICs, control and treated cells, either 5 h with 200 µM (Figure 8 and Figure 9) or with 2 mM of Na[*o*-COSAN] (Supplementary Figure S4), were calculated employing the multipeak curve fitting of the amide I region of the samples. We found a higher proportion of α-helix secondary structure in both GICs cell lines, GIC7 and PB88, induced by Na[*o*-COSAN], which inversely increased at high concentration of the compound (Table 1). Hen, Na[*o*-COSAN] might interact with proteins, especially with those having a high content of electrically positively charged side-chains, namely arginine, histidine, and lysine amino acids [65]. Therefore, the ratio amide I/amide II decreases with respect to the control cells in both GICs (Supplementary Table S1) [66]. Moreover, the secondary structure of amide I also changed by the treatment because the ratio α-helix/β-sheet decreased concerning their respective control cells.

By SR-FTIRM, it has been observed that Na[*o*-COSAN] interacts with proteins, inducing protein conformational changes in GICs. These data are consistent with its reported structure in complex with HIV PR that was determined at 2.15 Å resolution by protein crystallography, and it shows that two molecules of the [*o*-COSAN]^−^ bind to the hydrophobic pockets of HIV protease acting as a potent inhibitor of HIV protease [67].

#### 2.2.3. SR-FTIRM at the Lipids Region

**Figure 10:**
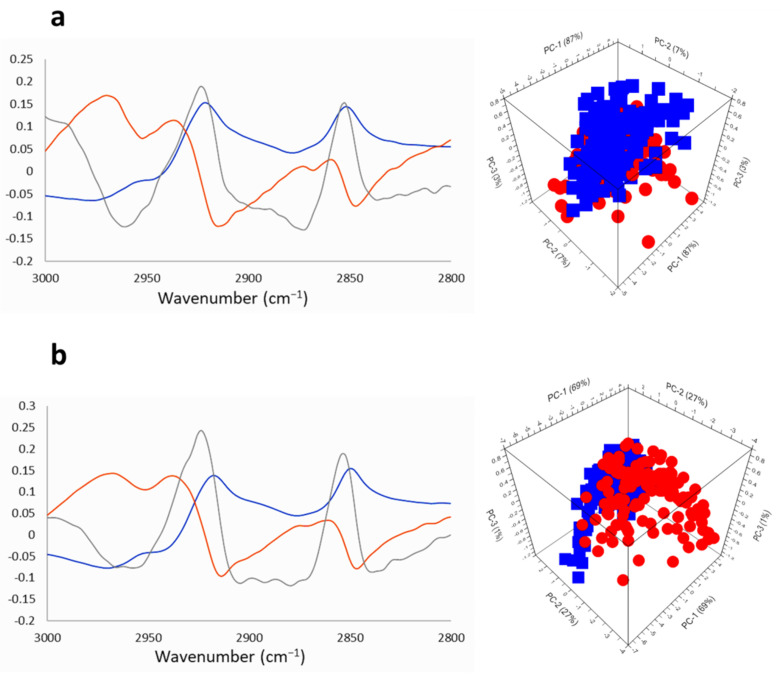
PCA and loadings within the lipid region (3000–2800 cm^−1^) of GICs samples treated with Na[*o*-COSAN] 200 µM: (**a**) GIC7 and (**b**) PG88 cells. Principal component loadings (blue PC-1, orange PC-2, grey PC-3) are shown on left panels. Right-panels show principal component analysis score plots of PC-1, PC-2, and PC-3 displaying the variance between GIC control cells (blue) and GIC treated cells (red).

The absorptions in the spectral range 3000–2800 cm^−1^ are from lipids vibrations (Figure 10a,b and Figure S6) [58]. Figure 10 displays the PCA corresponding to this region for GIC7 (Figure 10a) and PB88 cells (Figure 10b), both were performed from raw spectra corrected by EMSC of the GICs control vs. GICs treated with 200 µM of Na[*o*-COSAN] for 5 h. Significant differences were observed in PC-3, where the peaks at 2920 cm^−1^ and 2850 cm^−1^ from -CH_2_- asymmetric stretching and -CH_2_- symmetric stretching were slightly more intense in treated GIC7 with regard untreated control cells (Figure 10a). Furthermore, the frequency of the C_cluster_-H vibration at 3030 cm^−1^ showed a slight interaction with proteins or DNA. The ratios between 2960 cm^−1^ (-CH_3_ asymmetric stretching) and 2921 cm^−1^ (-CH_2_- asymmetric stretching) peaks were calculated (Supplementary Table S2) and in treated GIC7 increased concerning GIC7 control. In contrast, the absorbance ratios among the 2960 cm^−1^ (-CH_3_ asymmetric stretching) and 2921 cm^−1^ (-CH_2_- asymmetric stretching) of treated PG88 cells decreased noticeably concerning untreated control cells (Supplementary Table S2).

Finally, the lipids unsaturation index was calculated for both GICs by dividing the integral of the 3010 cm^−1^ signal (-HC=CH- stretching) by the integral of 2850 cm^−1^ signal (-CH_2_- symmetric stretching) (Supplementary Table S3) [43]. A partial reduction of lipid double bonds was observed in treated GIC7 concerning control cells, which is inversely proportional to Na[*o*-COSAN] concentration, while the unsaturation index of PG88 cells was also slightly affected by the treatment. More studies are needed to understand the lipid changes induced by Na[*o*-COSAN] in GICs and analyze in detail the consequences on specific cellular functions. 

### 2.3. Na[o-COSAN] Induces DNA, Proteins, and Lipids Changes on GICs

**Figure 11:**
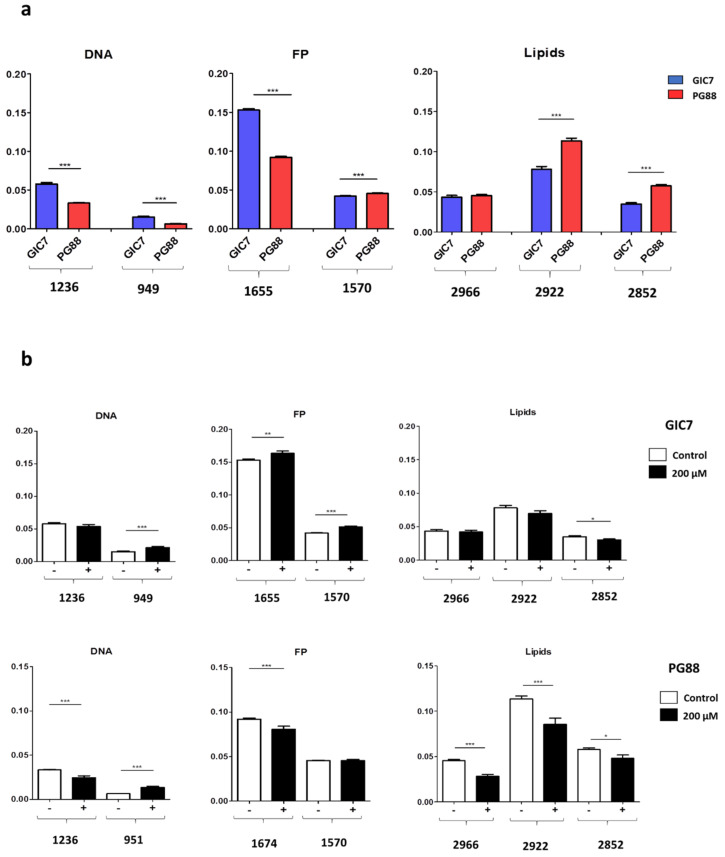
Comparison between GIC7 and PG88 analyses by SR-FTIRM- Histogram plots are representative from DNA, protein, and lipid peaks: (**a**) comparison between untreated GIC7 and PG88 cells and (**b**) comparison between treated cells with 200 µM Na[*o*-COSAN] 5 h with regard the respective untreated control cells (black and white columns, respectively), upper part GIC7 and PG88 at the bottom part. Significant differences by *t*-test, * *p* < 0.05; ** *p* < 0.01; *** *p* < 0.001 are pointed out at the corresponding peaks.

Since both GICs belong to non-mutated and non-G-CIMP (G-CIMP-) subtypes termed as proneural (GIC7) and mesenchymal (GP88), we explored DNA, proteins, and lipids differences between them (Figure 11a and Supplementary Table S4). SR-FTIRM micro-spectroscopy showed basal differences within DNA spectra, which involved 1236 cm^−1^ (Student’s *t*-test, *p* < 0.0001) and 949 cm^−1^ (Student’s *t*-test, *p* < 0.0001) peaks; 1655 cm^−1^ (Student’s *t*-test, *p* < 0.0001) and 1570 cm^−1^ (Student’s *t*-test, *p* < 0.0001) at the protein region; and 2922 cm^−1^ (Student’s *t*-test, *p* < 0.0001) and 2852 cm^−1^ (Student’s *t*-test, *p* < 0.0001) at the lipids area. These results suggested that these phenotypic differences might be involved in Na[*o*-COSAN] EC_50_ different responses exerted between GIC7 (95.87 µM) and PG88 (83.2 µM) cells. Therefore, the potential of SR-FTIRM to detect simple biochemical components might be used to stratify cells by their relative contents of lipids, glycogen, proteins, and other components, and this specific signature could predict therapy response. Indeed, ATR-FTIR spectroscopy has analytical capabilities for cancer diagnosis and is able to distinguish between healthy controls and brain cancer at sensitivities and specificities above 90%, and differentiate several types of brain tumors with accuracies >80% [68].

In addition, we analyzed by comparison spectroscopic differences between Na[*o*-COSAN] treated and untreated cells and we found important changes in biomolecules in both GIC7 and PG88 cell lines (Figure 11b and Supplementary Table S5), including the proteins’ secondary structure increasing the tendency to α-helix, lipid modifications, and DNA chains, either in GIC7 or PG88 cells. Some differences appeared specifically in GIC7 or PG88 cells. Indeed, we observe that Na[*o*-COSAN] induced an increase in β-sheet secondary structure of proteins in PG88 that was not observed in GIC7. 

The most significant DNA changes induced in treated GIC7 concerning untreated cells were at 949 cm^−1^ frequency (Student’s *t*-test, *p* = 0.0004), whereas in treated PG88 cells concerning untreated cells were at 951 cm^−1^ (Student’s *t*-test, *p* < 0.0001) and at 1236 cm^−1^ (Student’s *t*-test, *p* = 0.0002). Within proteins, the treatment increased 1655 cm^−1^ (Student’s *t*-test, *p* = 0.0033) and 1570 cm^−1^ (Student’s *t*-test, *p* < 0.0001) bands in GIC7, whereas in PG88 we did not find changes in these bands, and the one at 1674 cm^−1^ decreased significantly by the treatment (Student’s *t*-test, *p* = 0.0008). Moreover, lipids’ changes were observed mainly in PG88 cells at 2966 cm^−1^ (Student’s *t*-test, *p* < 0.0001) and 2922 cm^−1^ (Student’s *t*-test, *p* = 0.0008) frequencies.

GBM exhibits a high degree of phenotypic heterogeneity that molecularly corresponds to different gene expression signatures. We uncover large genetic diversity in primary and recurrent aggressive glioblastomas. However, all these diverse tumors map onto a common path of early tumorigenesis where characteristic driver mutations are acquired by losses or gains of (parts of) chromosomes [69]. In that scenario, BNCT therapy in recurrent glioblastomas has emerged as a novel therapeutic strategy, since the benefit of chemotherapy, the most frequently applied treatment in this stage, has limited improvement in survival and classical radiotherapy to treat recurrences is controversial [70]. However, it has been reported that after tumor-selective BNCT, recurrence was inevitable and hence the development of boron agents was important for the success of BNCT [71]. Herein, we have shown in two different GICs that the uptake of anionic inorganic small molecule cobaltabis(dicarbollide), [*o*-COSAN]^−^ can damage essential structures of cells, mainly with the lipids compartment being the most affected.

To demonstrate the effectiveness of [*o*-COSAN]^−^ to BNCT treatment is needed, more laboratory research combined with properly controlled trials investigating the in vivo assessment of the tumor-to-blood ratio and the [*o*-COSAN]^−^ concentration at tumor tissue, which had to be at least three times greater than in the normal brain [72]. Moreover, we have to find out if [*o*-COSAN]^−^ has low toxicity and both the tumor/brain and tumor/blood boron ratios are greater than 1, as has been demonstrated in in vivo experiments for this kind of compound.

GICs, which are functionally defined by their ability to self-renew and to initiate tumor formation in vivo, are responsible for the perpetuation of the tumor and are conveniently used to perform 3D cultures systems that recapitulate the complex reality of glioblastoma better. Since there are some strict requirements before using the boron drugs for BNCT application, such as their toxicity, solubility, and dosage accumulation in the cancer cells, we are currently using normal cerebral organoids. These form smooth, 3D spheroid structures, which are a good substrate to support the invasive growth of tumor spheres by generating projections of tumor mass beyond the boundaries of the organoid combining normal and tumor cells. However, the basic knowledge about pharmacokinetics and toxicity of boron compounds has been achieved by broad study of boron carriers for BNCT that can be useful for the development of boron compounds for cancer therapy. Here we afforded the two principal requirements: Na[*o*-COSAN] has a high GICs uptake without toxicity and rapid cell clearance but enough persistence to expect successful BNCT. Further work must afford the complementarity of in vitro and in vivo studies to yield significant insight into cell biological questions. Thus, in vitro culture studies and in vivo studies can be complementary, and together can yield significant insight into cell biological questions. Studies on 3D spheroids and in vivo in the small invertebrate C. elegans are undergoing and will be published as soon as possible.

## 3. Materials and Methods

### 3.1. Reagents

The NaCl was purchased from Sigma–Aldrich (Darmsdadt, Germany), whereas the cationic exchanging resin used (Amberlite IR120, H form) was purchased from Acros Organics (Geel, Belgium) and the hydrochloric acid (37%) was purchased from Labbox (Premià de Dalt, Spain).

RNase A (Sigma), which corresponds to a reagent of 0.02mg/mL PI (Sigma), and 0.1% (*v*/*v*) Triton X-100.

### 3.2. Chemistry

Cs [3,3′-Co(1,2-C_2_B_9_H_11_)_2_], Cs[*o*-COSAN], was synthesized from 1,2-*closo*-C_2_B_10_H_12_ from Katchem Spol.sr.o (Kralupy nad Vltavou, Czech Republic) as reported in the literature [48]. The [Na·2.5H_2_O][3,3′-Co(1,2-C_2_B_9_H_11_)_2_], Na[*o*-COSAN], was obtained by means of cationic exchange resin from Cs[*o*-COSAN] following the previously described procedure [73].

### 3.3. Cell Culture and Treatments

Both GICs belong to non-mutated and non-G-CIMP (G-CIMP-) subtypes. Figure 1 displays the optical microscopy images in the medium with and without laminin coated surface. GICs grow as neurospheres in non-laminin coated plates and in adherence in laminin coated surface. Cells placed on 7,5 mg/mL laminin-coated plates (Sigma, St. Louis, MO, USA) were maintained in a complete Neuronal Stem Cell (NSC) medium at 37 °C in a humidified 5% CO_2_ and 5% O_2_ atmosphere (hypoxia conditions) to simulate brain microenvironment (Heracell 150i incubator). NSC medium was constituted by Dulbecco’s Modified Eagle Medium and Nutrient Mixture F-12,DMEM/F12, (Gibco, Thermo Fisher Scientific, Inc., Waltham, MA, USA) supplemented with N_2_ (GIBCO), 4,5% glucose (Sigma, Merck KGaA, Darmstadt, Germany), 1M Hepes (Sigma), 2% BSA (Sigma) basic fibroblast growth factor 20 ng/mL (Gibco), and epidermal growth factor 20 ng/mL (Gibco).

GICs were treated with 200 µM or 2mM of Na[*o*-COSAN] to study the uptake of the anionic small molecule [*o*-COSAN]^−^ and its effects on cell function.

Glioma initiating cells (GICs), proneural GIC7 (a kind gift from Dr. Marta María Alonso, Department of Pediatrics, Clínica Universidad de Navarra, University of Navarra, Pamplona, Spain) [74], and mesenchymal PG88 cells were obtained from human GBM specimens as described previously [46,75].

### 3.4. Samples Preparation for SR-FTIRM Measurements

To analyze the interaction of the anionic small molecule [*o*-COSAN]^−^ with GICs, we seeded 100,000 cells in 12-well plates, containing a glass treated 4 h with UV light and coated with laminin (Sigma) at 1.5% in Dulbecco’s Phosphate Buffered Saline, DPBS (Gibco). GICs were incubated for 48 h in 1 mL of fresh NSC prior to add the Na[*o*-COSAN] and incubate 5 h in hypoxic conditions. Next, the medium was removed and the wells washed with DPBS and fixed 20 min with 4% of PFA in PBS followed by Millipore water washes. 

When cells grew on neurospheres, the wells were centrifuged at 700 rpm 4 min, washed, and disaggregated on 5 mL of DPBS, prior to fixing cells and placing the pellet on the CaF_2_ infrared windows to be analyzed. 

### 3.5. SR-FTIRM Measurements

FTIRM experiments in transmission mode were performed at MIRAS beamline of ALBA synchrotron light source (Barcelona, Spain) using a Hyperion 3000 Microscope coupled to a Vertex 70 spectrometer (Bruker, Karlsruhe, Germany) and equipped with a Mercury-Cadmium-Telluride (MCT) 50 µm detector. The synchrotron infrared light is focused on the sample using a 36× Schwarzschild objective (NA = 0.52) coupled to a 36× Schwarzschild condenser. The spectra were recorded through a 10 × 10 µm^2^ masking aperture size with a spectral resolution of 4 cm^−1^, within the Mid-IR spectral range of 4000–800 cm^−1^, and a co-addition of 100 scans per spectrum to improve the signal-to-noise ratio. Background spectra were collected every 5 cell measurements from a clear area that does not contain a sample. Single cells were mapped at 3 × 3 µm^2^ resolution using 1.5 µm distance between points.

### 3.6. FTIRM Software & Data Analysis

The OPUS 7.5 software (Bruker) was employed for data acquisition, while multivariate analysis was performed using the Unscrambler X10.3 software (CAMO Process AS, Oslo, Norway).

Prior to principal component analysis (PCA), extended multiplicative signal correction (EMSC) was applied on the raw spectra, following previous protocols [76,77]. Standard normal variate (SNV) normalization was applied in the following regions: the fingerprint (1800–1000 cm^−1^) and the lipids (3000–2800 cm^−1^) regions. Second derivative spectra were calculated using the Savitzky–Golay algorithm (third polynomial order; 9 smoothing points) for the DNA region (1350–900 cm^−1^) and unit vector normalized.

The deconvolution of the Amide I signals was made by calculating the average spectra for each sample in the Amide I region (1700–1600 cm^−1^), applying the linear baseline correction, and then the spectra were unit vector normalized. Once corrected, second derivative spectra were calculated using the Savitzky–Golay algorithm (third polynomial order; 9 smoothing points). The peaks of each protein secondary structure were assigned using the second derivative minimum values at the following values: 1610 cm^−1^ for sidechain, 1630 cm^−1^ for β-sheet, 1652 cm^−1^ for α-helix, 1682 cm^−1^ for β-turn, and 1690 cm^−1^ for β-sheet antiparallel [64]. Using these wavenumbers, each peak was assigned to a Gaussian function. The parameters of each function were calculated using Solver extension of Microsoft Excel by reducing the square errors of the sum of the Gaussians with the experimental spectra.

### 3.7. Kinetic Assay

For kinetic assay, 5000 GICs were seeded in each well of a 96 multi-well plate in a complete NSC medium for 48 h at 37 °C in 5% CO_2_ and 5% O_2_ atmosphere, before incubation with 200 µM of Na[*o*-COSAN] in fresh NSC medium and sampling supernatant at 0 min, 15 min, 30 min, 45 min, and 1 h to lyse cells in each well with 0.2% of DMSO in PBS, to evaluate Na[*o*-COSAN] uptake (Figure 2a) by the spectrometer Synergy (BioTek, Agilent Technologies, Santa Clara, CA, USA) at 282 nm. Cells with or without Na[*o*-COSAN] and wells with Na[*o*-COSAN] and medium without cells were used as negative and positive controls, respectively.

### 3.8. PrestoBlue Assay

PrestoBlue is a resazurin-based membrane permeable solution, which, upon reduction by living cells, forms a red fluorescent compound called resorufin via mitochondrial enzymes of viable cells in the tested systems. Consequently, the reagent exhibits a change in color, and a shift in its fluorescence, which is quantitatively measurable providing information on the cell viability. For the experiment, 5000 GICs, GIC7 and PG88, were seeded in each well of a 96 multi-well plate in complete culture medium. The cells were incubated 48 h at 37 °C under 5% CO_2_ and 5% O_2_. Next, the medium was aspirated and Na[*o*-COSAN] in fresh medium was added to achieve the final tested concentrations [µM]: 50, 75, 100, 125, 150, 175, 200, 250, and 300, added to each well in triplicates. Control cells that contained only cells in medium without Na[*o*-COSAN] were also incubated at 37 °C under 5% CO_2_ and 5% O_2_ for 48 h. Next, 10 µL of PrestoBlue 10X (Life Technologies, Carlsbad, CA, USA) in 90 µL of fresh NSC medium was added and incubated at 37 °C for 2 h. Absorbance (Abs) was recorded at wavelength of 570 nm and 600 nm by the Synergy (BioTek, Agilent Technologies). Data was normalized according to the manufacture protocol.

### 3.9. Cytotoxicity and Half Maximal Effect Concentration (EC_50_)

Cytotoxicity and EC_50_ were determined by PrestoBlue assays according to Nowak et al. and Organization for Economic Cooperation and Development (OECD) protocol [78]. The absorbance of the control sample (untreated cells) represented 100% cell viability. Cell viability (%) was calculated as follows: [(A570 sample−A570 blank)/(A570 control−A570 blank) × 100], and cytotoxicity (%) was determined with respect to 100% of cell viability of the control (untreated cells). The results were presented as mean ± standard deviation (SD)/standard error of the mean (SEM) in Figure 2b. The value of EC_50_ was determined from curves according to OECD protocol.

### 3.10. Cell Cycle Analysis

Cell cycle was analyzed by flow cytometry after mark with propidium iodide (PI) the cells. GIC7 and PG88 cells were treated with 200 µM and 2 mM of Na[*o*-COSAN] in complete medium for 5 h and after recovery 43 h later. Following treatment, cells were harvested, washed with PBS, and fixed with 70% ethanol at 4 °C during 2 h. Next, the ethanol-suspended cells were centrifuged 5 min at 200 rpm. The ethanol was decanted thoroughly and the cell pellet was suspended in 5 mL PBS and centrifuged 5 min at 200 rpm. Finally, cell pellet was suspended in 500 μL PI/Triton X-100 staining solution with RNase A (0.02mg/mL PI (Sigma), 0.1% (*v*/*v*) Triton X-100 (Sigma) and 2mg DNase-free RNase A (Sigma) and incubated 15 min at 37 °C. Cycle analysis was performed using flow cytometry system. FlowJo software(v 10.0) was used to determine the proportions of cells in different cell stages of cell cycle progression (Sub G_0_, G_0_/G_1_, S, and G_2_/M phases).

### 3.11. Microscopic Observations of Cell Morphology

GIC7 and PG88, GICs, 5000 cells/well seeded in 96 multi-well plates, at 37 °C under 5% CO_2_ and 5% O_2_ were analyzed after the addition of Na[*o*-COSAN] 200 µM of NSC fresh medium. Cells without Na[*o*-COSAN] were used as controls. Cellular morphological and density changes were observed under phase contrast microscope (Olympus) and photographed (Figure 3a,b) to analyze the treated cells recovery. Photographic material was obtained before adding Na[*o*-COSAN] (control with vehicle), after 5 h incubation with Na[*o*-COSAN] and after incubating the previous rinsed cells during 43 h with fresh NSC medium.

### 3.12. Statistical Analysis

Two-tailed Student’s *t* test (to compare two experimental groups) or an ANOVA (to compare three or more groups) were performed for data analysis using GraphPad Prism (GraphPad Software). Bonferroni post-test was performed to compare replicate means. For all statistical methods, *p* < 0.05 was considered significant.

## 4. Conclusions

In this work infrared micro-spectroscopy has been successfully used to detect the cellular uptake of the Na[*o*-COSAN] and interactions between [*o*-COSAN]^−^ and biomolecules (lipids, proteins and DNA) inside cells.

SR-FTIRM can be applied to the uptake detection of boron clusters (boranes, carboranes, and metallacarboranes) because these compounds’ families display a strong and characteristic ν(B-H) frequency in the infrared range 2.600–2.500 cm^−1^ in which no other frequencies of organic compounds appear. The observed interactions between [*o*-COSAN]^−^ and biomolecules (lipids, proteins and DNA) that were reported on a chemical scale have been detected inside cells by means of SR-FTIRM.

The small molecule Na[*o*-COSAN], localized close to the cell’s nucleus, induces proteins’ conformational changes and spectral changes of the DNA region in both GIC cell lines similar to the changes induced by other metal-based compounds like cisplatin that disrupt the double helix base pairing, suggesting that Na[*o*-COSAN] is a promising agent for BNCT of glioblastoma. Furthermore, our studies also show that mesenchymal PG88 cells that are more resistant than proneural GIC7 cells to conventional radiotherapy have a lower EC_50_ Na[*o*-COSAN] and a higher uptake of the compound compared to GIC7 cells, suggesting a new resource to fight against resistant glioblastoma cells.

Furthermore, SR-FTIR micro-spectroscopy analysis differentiates the two phenotypes of glioma initiating cells (GICs), mesenchymal and proneural GICs, mainly by DNA spectra, protein conformation and lipidic components, which were differently modified by Na[*o*-COSAN]. Interestingly, specific changes in lipid 2966 cm^−1^ and 2922 cm^−1^ frequencies are induced in radio resistant mesenchymal cells PG88, the most efficient in capturing the compound. Although more experiments are needed including representative GICs from glioblastoma phenotypes, these results suggest that SR-FTIRM is useful to acquire a drug and radio-resistance profiling of glioblastoma cells. Moreover, the high uptake of Na[*o*-COSAN] with rapid clearance but still enough persistence is expected to allow successful BNCT application to fulfil the requirements for boron-containing agents. Although SR-FTIRM is able to distinguish different subtypes of GICs, further tissue-based studies are needed to determine whether FTIR can stratify subtypes of cerebral glioma by identifying differences in DNA, proteins, and lipids molecules.

## Figures and Tables

**Table 1:** Ratio of the α-helix and β-sheet Integrated spectra obtained from the deconvolution of Amide I from GICs.

Sample	Ratio ∫α/∫β	ΣError^2^
GIC7 Control	2.37	4.9 × 10^−5^
GIC7 200 µM	1.26	3.8 × 10^−4^
GIC7 2 mM	2.54	2.2 × 10^−4^
PG88 Control	10.62	3.3 × 10^−4^
PG88 200 µM	7.19	7.4 × 10^−4^
PG88 2 mM	8.00	6.6 × 10^−4^

## Data Availability

Not applicable.

## Supplementary Materials

The following are available online at www.mdpi.com/article/10.3390/ijms22189937/s1.
