# Supplementary material for: Synchrotron-Based Fourier-Transform Infrared Micro-Spectroscopy (SR-FTIRM) Fingerprint of the Small Anionic Molecule Cobaltabis(dicarbollide) Uptake in Glioma Stem Cells
Source: Int J Mol Sci. 2021 Sep 14;22(18):9937. doi: 10.3390/ijms22189937 (PMC8466526; doi:10.3390/ijms22189937)
Supplement: Supplementary file 1 [file ijms-22-09937-s001.zip › ijms-1342681-supplementary.pdf]

SUPPORTING INFORMATION

Synchrotron-Based Fourier-Transform Infrared Micro-SPECTROSCOPY (SR-FTIRM) Fingerprint of the Small Anionic Molecule Cobaltabis(dicarbollide) Uptake in Glioma Stem Cells

Miquel Nuez-Martínez [1,†], Leire Pedrosa [2,†], Immaculada Martinez-Rovira [3,4], Ibraheem Yousef [4], Diouldé Diao [2], Francesc Teixidor [1], Elisabetta Stanzani [5], Fina Martínez-Soler [6], Avelina Tortosa [6], Àngels Sierra [2,*], José Juan Gonzalez [2] and Clara Viñas [1,*]

[1] Institut de Ciència de Materials de Barcelona, ICMAB-CSIC, Campus Universitat Autònoma de Barcelona, 08193 Bellaterra, Spain; mnuez@icmab.es (M.N.-M.); teixidor@icmab.es (F.T.)
[2] Laboratory of Experimental Oncological Neurosurgery, Neurosurgery Service, Hospital Clinic de Barcelona—FCRB, 08036 Barcelona, Spain; lepedrosa@clinic.cat (L.P.); dioulde.dg@gmail.com (D.D.); jjgonzal@clinic.cat (J.J.G.)
[3] Ionizing Radiation Research Group (GRRI), Physics Department, Universitat Autònoma de Barcelona (UAB), Avinguda de l'Eix Central, Edifici C. Campus de la UAB, 08193 Cerdanyola del Vallès, Spain; Immaculada.Martinez@uab.cat
[4] ALBA-CELLS Synchrotron, MIRAS Beamline, Carrer de la Llum 2-26, 08290 Cerdanyola del Vallès, Spain; iyousef@cells.es
[5] Laboratory of Pharmacology and Brain Pathology, IRCCS Humanitas Research Hospital, 20089 Rozzano, Italy; stanzani.elisabetta@gmail.com
[6] Apoptosis and Cancer Unit, Department of Physiological Sciences, IDIBELL, Faculty of Medicine and Health Sciences, Universitat de Barcelona, 08907 L'Hospitalet del Llobregat, Spain; finamartinez@ub.edu (F.M.-S.); atortosa@ub.edu (A.T.)
* Correspondence: masierra@clinic.cat (À.S.); clara@icmab.es (C.V.)
† The authors contributed equally to the work.

INDEX

	Page
**Figure S1.** Average spectra (left) and the corresponding 2nd derivative of the average spectra at the DNA region (1350-900 cm^{-1}).	3
**Figure S2.** PCA and loadings in the DNA region (1350-900 cm-1) of GICs samples treated 5 h with Na[*o*-COSAN] 2Mm.	4
**Figure S3.** SR-IRFT average spectra of GICs samples in the fingerprint proteins region 1800-1350 cm−1 after 5 h Na[*o*-COSAN] 200 µM	5
**Figure S4.** GIC7 control vs GIC incubated with Na[*o*-COSAN] 2 mM: a) Fingerprint PCA and loadings of the GIC7 samples in the proteins region. b) Amide I deconvolutions. PG88 control vs PG88 incubated with Na[o-COSAN] 2 mM: c) Fingerprint PCA and loadings of the PG88 samples in the proteins region. d) Amide I deconvolutions.	6
**Figure S5.** Average spectra of GICs cell samples in the lipids región after incubated with Na[o-COSAN] 200µM.	7
**Figure S6.** PCA and loadings of GICs cell samples in the lipids region incubated with Na[COSAN] 2mM.	8
**Table S1**. Amide I / Amide II ratios of absorbance in GIC7 and PG88 samples.	9
**Table S2.** Ratios of 2960 cm^{-1} and 2921 cm^{-1} signals in GIC7 and PG88 samples.	9
**Table S3.** Ratios of 3010 cm^{-1} band integral and 2850 cm-1 band integral in GIC7 and PG88 samples.	10
**Table S4.** Statistical data from representative DNA, protein and lipid different peaks comparing GIC7 and PG88 cells.	10
**Table S5**. Statistical data from representative DNA, protein and lipid different peaks in 200 µm Na[o-COSAN] 5 h treated v.s. untreated GIC7 and PG88 cells.	11

Figure S1. Average spectra (left) and the corresponding 2nd derivative of the average spectra (right) at the DNA region (1350-900 cm^{-1}). **a)** GIC7 and **b)** PG88 treated with Na[*o*-COSAN] 200 µM (red line) and untreated respective controls (blue line).

a

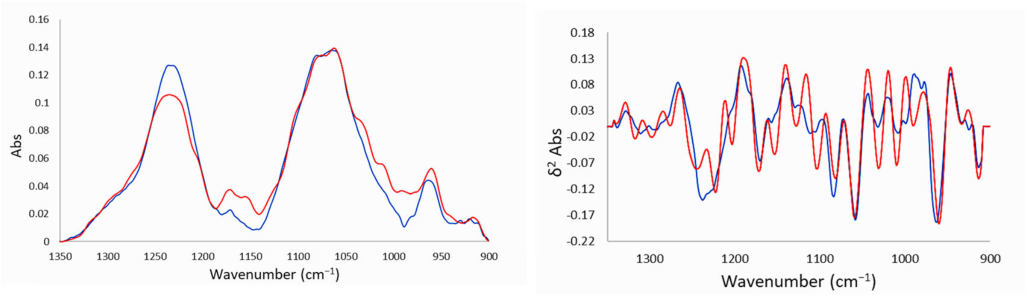

b

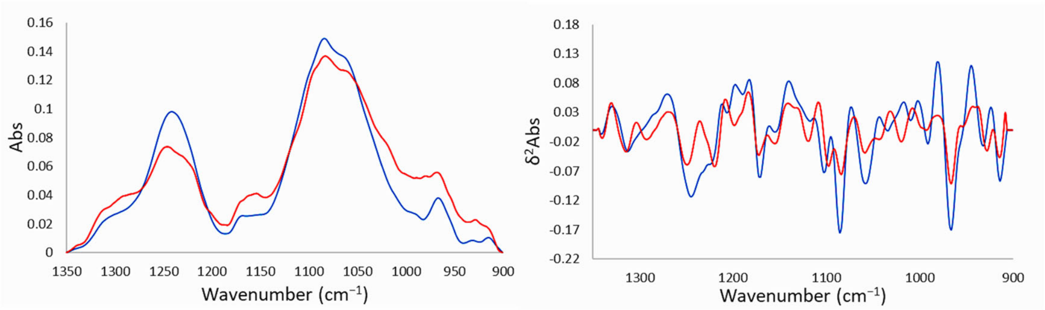

Figure S2. PCA and loadings in the DNA region (1350-900 cm-1) of GICs samples treated 5 h with Na[*o*-COSAN] 2mM: **a)** GIC7 cells control (light blue) *v.s.* GIC7 treated (blue) and **b)** PG88 cells control (light blue) *v.s.* PG88 treated (blue). Average spectra (left) and the corresponding 2nd derivative of the average spectra (right) at the DNA region are showed in the upper panels. Left-bottom panels of **a)** and **b)**: Principal components (blue PC-1, orange PC-2, grey PC-3). Right-bottom of **a)** and **b)**: Principal component analysis scores plot of PC-1, PC-2 and PC-3 displaying the variance between control and treated GICs.

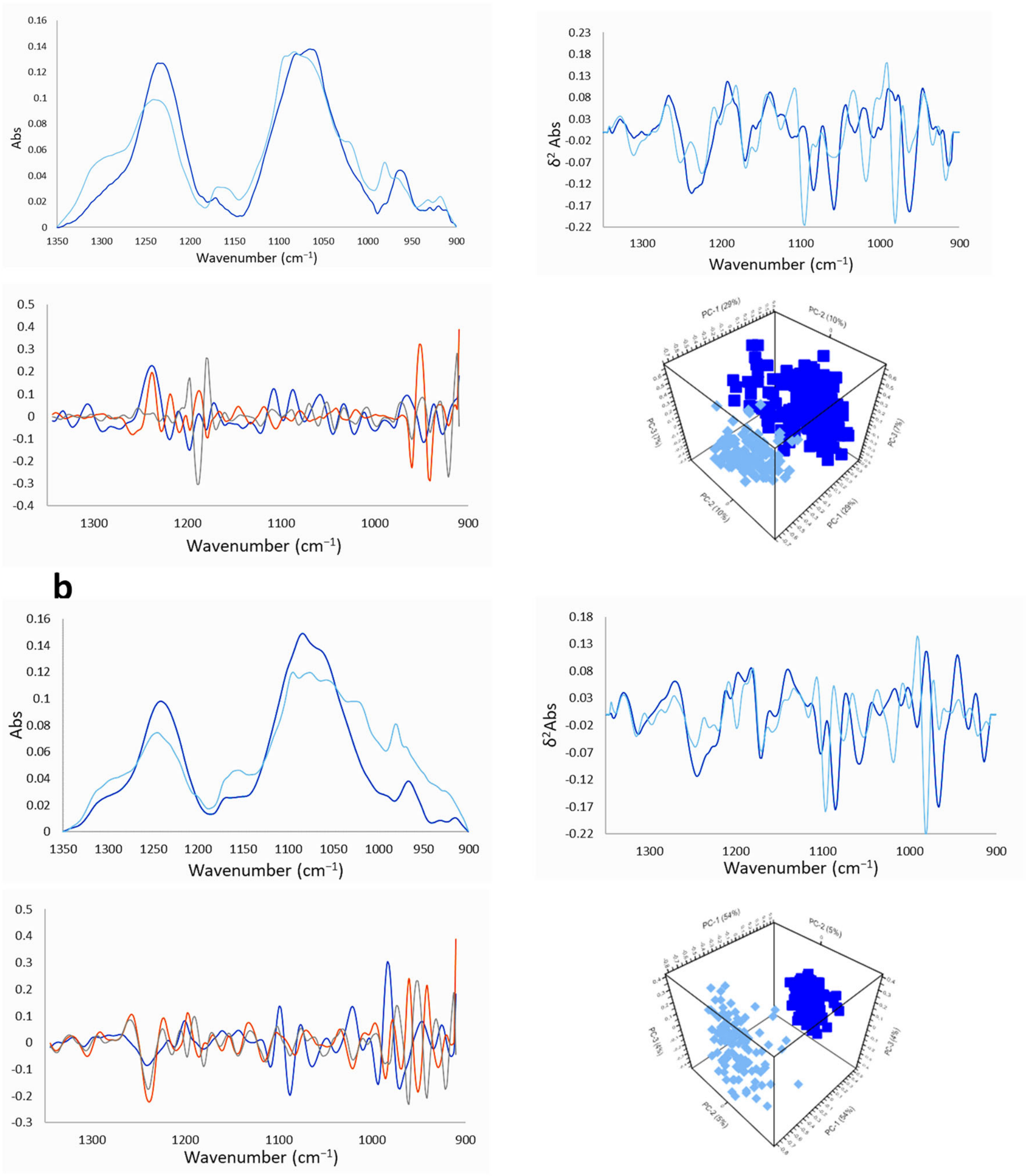

Figure S3. SR-IRFT average spectra of GICs samples in the fingerprint proteins region 1800-1350 cm−1 after 5 h Na[*o*-COSAN] 200 µM: a) GIC7 control (blue) vs GIC7 treated (red) and b) PG88 control (blue) vs PG88 treated (red).

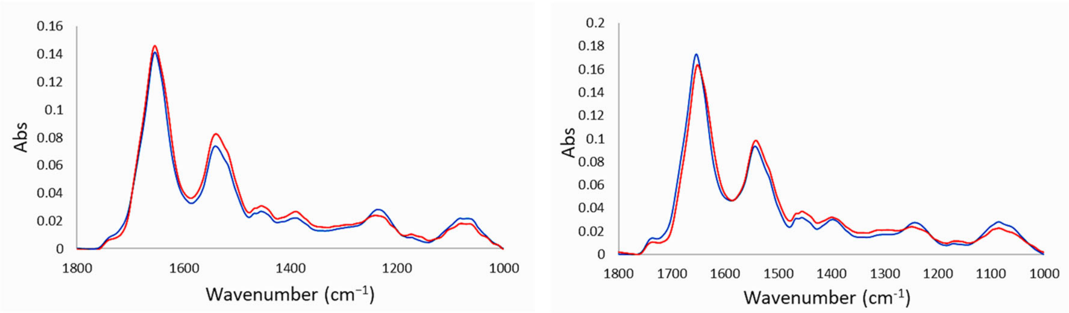

Figure S4. a) Fingerprint PCA and loadings of the GIC7 samples in the proteins region of SR-IRFT spectra. GIC7 control vs GIC incubated with Na[*o*-COSAN] 2 mM. b) Amide I deconvolutions for GIC7 2 mM sample and summary of GIC7 Control (in blue), GIC7 200 μM (in red dotted) and GIC7 2 mM (in black dotted). c) Fingerprint PCA and loadings of the PG88 samples in the proteins region of SR-IRFT spectra. PG88 control vs PG88 incubated with Na[*o*-COSAN] 2 mM. d) Amide I deconvolutions for PG88 2 mM sample and summary of PG88 Control (in blue), PG88 200 μM (in red dotted) and PG88 2 mM (in black dotted). The deconvolutions follow the following code: Amide I-experimental in dark blue, Sidechain in orange, β-sheet in grey, α-helix in blue, β-turn in green, β-sheet antiparallel in black and Amide I-calculated in red dotted.

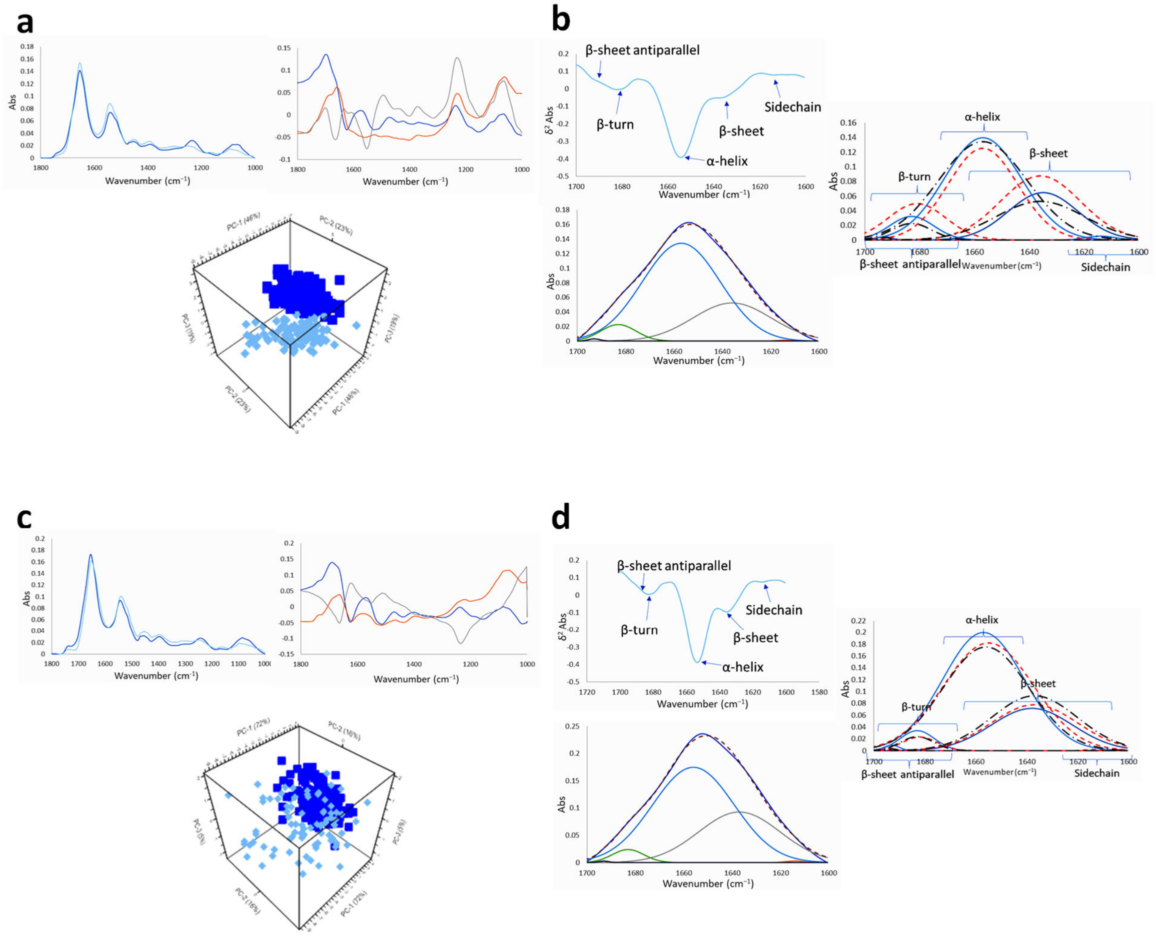

Figure S5. a) Average spectra of GICs cell samples in the lipids region. GIC7 control vs GIC7 incubated with Na[*o*-COSAN] 200µM. b) Average spectra of PG88 cell samples in the lipids region. PG88 control vs PG88 incubated with Na[o-COSAN] 200µM.

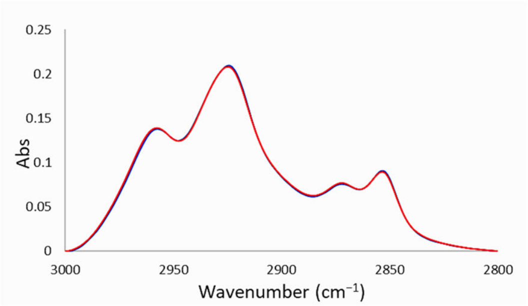

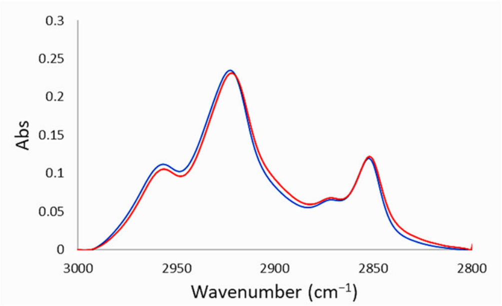

Figure S6. a) PCA and loadings of GICs cell samples in the lipids region. GIC7 control vs GIC7 incubated with Na[*o*-COSAN] 2mM. b) PCA and loadings of PG88 cell samples in the lipids region. PG88 control vs PG88 incubated with Na[COSAN] 2mM.

a

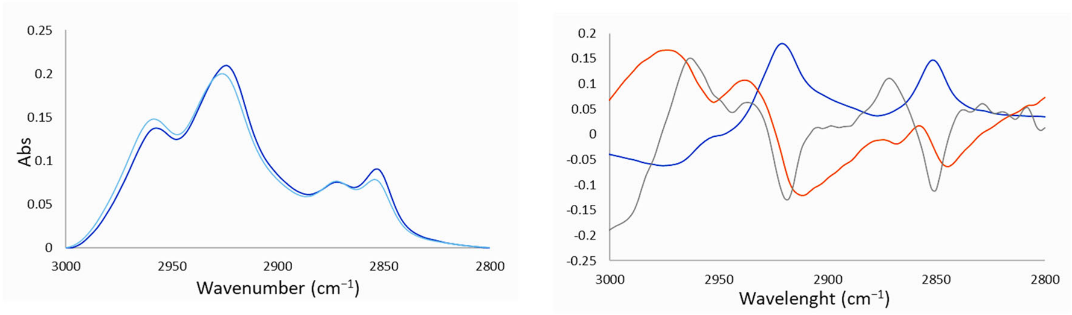

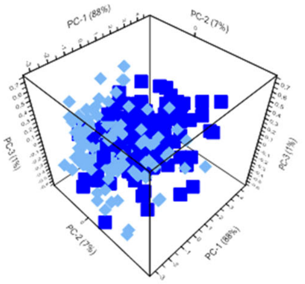

b

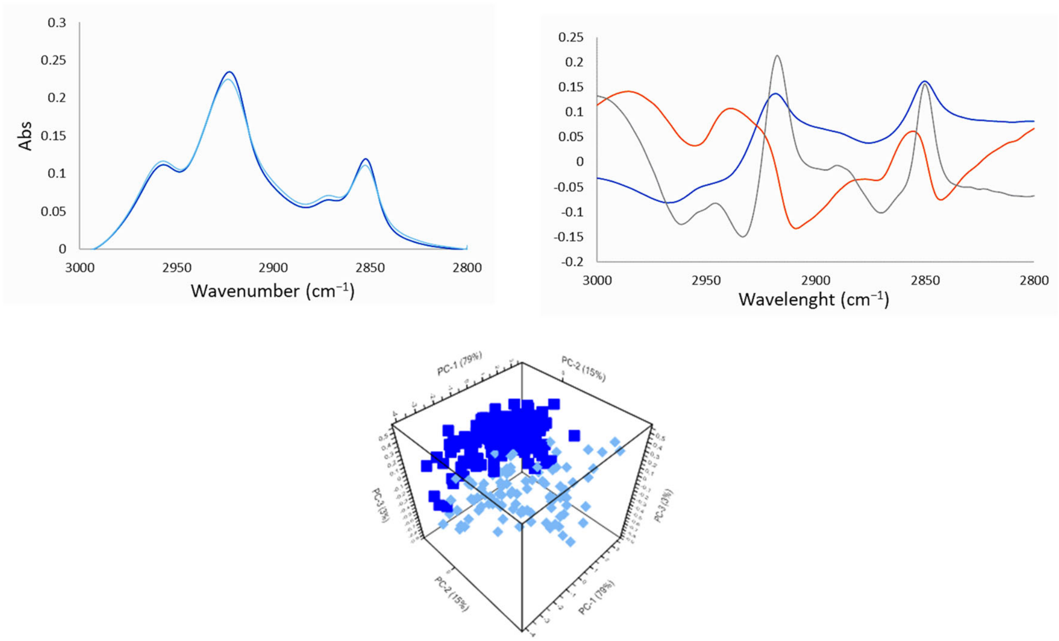

Table S1. Amide I / Amide II ratios of absorbance in GIC7 and PG88 samples.

Sample	Ratio (Amide I/AmideII)
GIC7 Control	1.81±0.16
GIC7 200 μM	1.76±0.25
GIC7 2 mM	1.67±0.20
PG88 Control	1.76±0.12
PG88 200 μM	1.52±0.14
PG88 2 mM	1.44±0.14

Table S2. Ratios of 2960 cm^{-1} and 2921 cm^{-1} signals in GIC7 and PG88 samples.

Sample	Ratio (aCH_3/aCH_2)
GIC7 Control	0.67±0.08
GIC7 200 μM	0.70±0.09
GIC7 2 mM	0.77±0.06
PG88 Control	0.46±0.06
PG88 200 μM	0.40±0.06
PG88 2 mM	0.47±0.07

Table S3. Ratios of 3010 cm^{-1} band integral and 2850 cm^{-1} band integral in GIC7 and PG88 samples.

Sample	Ratio ∫-HC=CH- St./∫-CH_2- Sym.
GIC7 Control	0.080±0.034
GIC7 200 μM	0.047±0.028
GIC7 2 mM	0.013±0.017
PG88 Control	0.051±0.014
PG88 200 μM	0.064±0.034
PG88 2 mM	0.030±0.018

Table S4. Statistical data from representative DNA, protein and lipid different peaks comparing GIC7 and PG88 cells.

	Molecule	λ	Significant? P < 0.05?	p-value	t, df	Mean Diff. (cnt vs treated)	95% CI of diff	R square
Unpaired t test GIC7 vs PG88	DNA	1236	***	P<0.0001	t=11.52 df=314	0.02453 ± 0.002129	0.02036 to 0.02870	0,2972
		949	***	P<0.0001	t=8.943 df=314	0.008683 ± 0.0009709	0.006780 to 0.01059	0,203
	FP	1655	***	P<0.0001	t=28.48 df=314	0.06093 ± 0.002139	0.05673 to 0.06512	0,7209
		1570	***	P<0.0001	t=5.404 df=314	-0.003433 ± 0.0006352	-0.004678 to -0.002188	0,08509
	Lipids	2966	ns	0,4725	t=0.7193 df=314	-0.001853 ± 0.002576	-0.006901 to 0.003196	0,001645
		2922	***	P<0.0001	t=7.090 df=314	-0.03537 ± 0.004988	-0.04514 to -0.02559	0,138
		2852	***	P<0.0001	t=9.398 df=314	-0.02271 ± 0.002417	-0.02745 to -0.01798	0,2195

Table S5. Statistical data from representative DNA, protein and lipid different peaks in 200 µm Na[*o*-COSAN] 5 h treated v.s. untreated GIC7 and PG88 cells.

Unpaired t test	Molecule	λ	Significant? P < 0.05?	p-value	t, df	Mean Diff. (cnt vs treated)	95% CI of diff	R square
GIC7	DNA	1236	ns	0,2495	t=1.154 df=295	0.004059 ± 0.003518	-0.002837 to 0.01096	0,004492
		949	***	0,0004	t=3.563 df=295	-0.006368 ± 0.001787	-0.009871 to - 0.002865	0,04125
	FP	1655	**	0,0033	t=2.966 df=295	-0.01048 ± 0.003532	-0.01740 to - 0.003553	0,02895
		1570	***	P<0.0001	t=8.648 df=295	-0.009467 ± 0.001095	-0.01161 to - 0.007321	0,2022
	Lipids	2966	ns	0,6499	t=0.4543 df=295	0.001549 ± 0.003410	-0.005135 to 0.008234	0,0006993
		2922	ns	0,1165	t=1.574 df=295	0.008471 ± 0.005380	-0.002074 to 0.01902	0,008333
		2852	*	0,0439	t=2.024 df=295	0.004800 ± 0.002372	0.0001512 to 0.009449	0,01369
PG88	DNA	1236	***	0,0002	t=3.756 df=273	0.008880 ± 0.002364	0.004245 to 0.01351	0,04912
		951	***	P<0.0001	t=6.544 df=273	-0.007144 ± 0.001092	-0.009284 to - 0.005005	0,1356
	FP	1674	***	0,0008	t=3.403 df=273	0.01136 ± 0.003339	0.004816 to 0.01790	0,04068
		1570	ns	0,9565	t=0.05455 df=273	0.00007323 ± 0.001342	-0.002558 to 0.002704	0,0000109
	Lipids	2966	***	P<0.0001	t=6.537 df=273	0.01729 ± 0.002645	0.01211 to 0.02248	0,1353
		2922	***	0,0002	t=3.811 df=273	0.02808 ± 0.007369	0.01364 to 0.04252	0,0505
		2852	*	0,0164	t=2.414 df=273	0.009733 ± 0.004032	0.001830 to 0.01764	0,0209
